# Linking Measures of Colony and Individual Honey Bee Health to Survival among Apiaries Exposed to Varying Agricultural Land Use

**DOI:** 10.1371/journal.pone.0152685

**Published:** 2016-03-30

**Authors:** Matthew Smart, Jeff Pettis, Nathan Rice, Zac Browning, Marla Spivak

**Affiliations:** 1 University of Minnesota, Department of Entomology, St. Paul, MN, United States of America; 2 USDA-ARS-Bee Research Lab, Beltsville, MD, United States of America; 3 Browning’s Honey Company, Jamestown, ND, United States of America; Federal University of Viçosa, BRAZIL

## Abstract

We previously characterized and quantified the influence of land use on survival and productivity of colonies positioned in six apiaries and found that colonies in apiaries surrounded by more land in uncultivated forage experienced greater annual survival, and generally more honey production. Here, detailed metrics of honey bee health were assessed over three years in colonies positioned in the same six apiaries. The colonies were located in North Dakota during the summer months and were transported to California for almond pollination every winter. Our aim was to identify relationships among measures of colony and individual bee health that impacted and predicted overwintering survival of colonies. We tested the hypothesis that colonies in apiaries surrounded by more favorable land use conditions would experience improved health. We modeled colony and individual bee health indices at a critical time point (autumn, prior to overwintering) and related them to eventual spring survival for California almond pollination. Colony measures that predicted overwintering apiary survival included the amount of pollen collected, brood production, and *Varroa destructor* mite levels. At the individual bee level, expression of *vitellogenin*, *defensin1*, and *lysozyme2* were important markers of overwinter survival. This study is a novel first step toward identifying pertinent physiological responses in honey bees that result from their positioning near varying landscape features in intensive agricultural environments.

## Introduction

Numerous stressors have been implicated as potentially contributing to losses of honey bee colonies (e.g. [1,2]) including such factors as: 1) poor diet brought on by inadequate and/or deficient nutritional resources [3–5], 2) exposure to environmental and in-hive pesticides [6–9], and 3) pests and pathogens [1,3,10–13]. In light of the fact that no single factor has been shown to occur in all cases of colony failure, a more nuanced view of the dynamics occurring inside and outside the hive has emerged–one in which multiple factors working together and/or at various times of the year, may lead to colony failure [8,9,13–15].

Nutrition and forage, specifically, are emerging as important factors that may influence the expression of other stressors such as viruses and *Varroa* mites that ultimately impact the health, productivity, and survival of honey bee colonies [4,16,17]. Honey bees are dependent on the adequate availability and collection of pollen to meet most of their dietary needs, and are completely dependent on pollen availability for brood-rearing [18]. By consuming fresh or stored pollen (i.e. bee bread), and engaging in trophallaxis among nestmates, bees acquire the amino acids, lipids, vitamins, and minerals necessary for growth and development. Colonies maintain a modest, yet consistent (around 1 kg), store of pollen in the hive throughout the growing season which is enough to last for approximately one week in the event of a prolonged pollen dearth [19]. Pollen balance is central to the growth and sustainability of colonies, and affects many downstream processes such as brood-rearing and behavioral development of workers, and interactions between diet, nutrition, and disease and/or immune system status [16,20].

At the individual bee level, protein nutrition affects maturation and longevity, overwintering survival, nutritional physiology, and immune competence [4,21,22]. Laboratory studies have identified several nutritional and immunological indices relating the health of individual bees, including (in response to improved nutrition) an increase in the size and protein content of the brood-food producing hypopharyngeal glands [16,23–25], higher levels of the storage protein vitellogenin [4,5,22], higher abdominal lipid stores [26,27], and variable immune system responses [5,27].

Few studies have bridged the gap between individual bee measures and colony level phenomena, and related these combined measures to ultimate outcomes in large-scale, migratory beekeeping operations. Dainat et al. [28] found three factors, deformed wing virus, *Varroa destructor* mites, and *Nosema ceranae* were elevated in dying colonies, and expression of *vitellogenin* in individual bees within those colonies was decreased. In this case, however, the colonies were not treated to control *Varroa*. Sagili and Pankiw (2007) found that individual bees treated with a trypsin inhibitor contained hypopharyngeal glands with decreased protein content, and colonies containing such individuals subsequently reared less brood [25]. Other research has indicated that treatment of individual bees with a lipid biosynthesis inhibitor accelerates maturation and precocious foraging efforts of those bees when placed in single-cohort colonies [26], while yet others have shown that nutritionally stressed larvae become poor foragers and dancers as adult workers [17].

We have shown that when diseases and parasites were controlled in bee colonies, and the effects of pesticide exposure were ruled out, the area of uncultivated land surrounding apiaries (Table 1) had a significant impact on the survival of commercial honey bee colonies in the Northern Great Plains region [29]. We suggested that under these conditions, the large-scale landscape around bee colonies influenced colony survival, specifically through the differential forage and nutrition via floral resource availability to colonies in surrounding lands over the growing season [29].

**Table 1:** Apiary land use (mean 2010–12 hectares) by site.

Site	Uncultivated forage	Cultivated forage	Wetlands	Corn	Soybeans	Wheat
A	2239	32	221	139	286	127
B	1670	16	485	238	557	198
C	970	159	39	512	1006	333
D	928	27	436	150	1025	598
E	537	56	121	568	1249	512
F	380	3	318	580	1450	413

Uncultivated forage included pasture, conservation reserve program land, grassland, hay land, fallow land, flowering woody plants, and roadside ditches. Cultivated forage included canola, sunflower, and alfalfa. Wetlands included seasonal and permanent wetlands, and cattails.

Here, we present measures of colony and individual bee health from the same six apiary sites in the same land use context, over a three-year period. Twenty-four different colonies per each of the six sites were assessed each year. Given our previous findings of land use impacts on survival [29], our objectives were to:

1. Determine if land use surrounding the apiary sites had specific and quantifiably different effects on colony and individual bee health measures; and
2. Use these markers of colony and individual bee health to develop statistical models that help predict the survival of honey bee colonies.

## Results

### Colony level measures

#### Survival and productivity of colonies

The survival of colonies within sites was statistically related to the large-scale land use surrounding apiaries. Colonies in site F experienced the lowest survival, particularly in 2010 and 2011, while colonies in site A experienced the greatest survival in all years (Table 2). The number of surviving colonies in each site (6 sites) and year (3 years) was used as the response variable for statistical modeling of colony and individual bee measures. A similar pattern was observed for honey production, though greater year-to-year variation occurred (S1 Table) related to climatic factors.

**Table 2:** Annual survival, 2010–2013. Survival is the percentage of colonies surviving May-March of each year out of the 24 assessed colonies per site.

Year	Site	Number colonies surviving (Percent total)
	A	20 (83)
	B	17 (67)
2010	C	19 (79)
	D	20 (83)
	E	18 (75)
	F	12 (50)
	A	20 (83)
	B	17 (71)
2011	C	18 (75)
	D	18 (75)
	E	18 (75)
	F	12 (50)
	A	21 (88)
	B	19 (79)
2012	C	17 (71)
	D	18 (75)
	E	16 (67)
	F	17 (71)

#### Population of bees and brood

Colony population, measured in combs of adult bees every six weeks year round, peaked in July-September and declined over the winter until foraging was resumed during March almond pollination of each year. Populations of adult bees were at their lowest in January of each year. The seasonal variation in colony adult bee population led to significant interactions between site and date (S1 Fig: F_100,2383_ = 2.58, p<0.0001). Data only for the month of September were used for statistical modeling purposes because colony health in early fall is a critical for overwintering success. In September, a significant interaction also was found between site and year (S2 and S3 Tables) but only in 2010, with site E having a larger population of bees than site B at that time.

**Fig 1:**
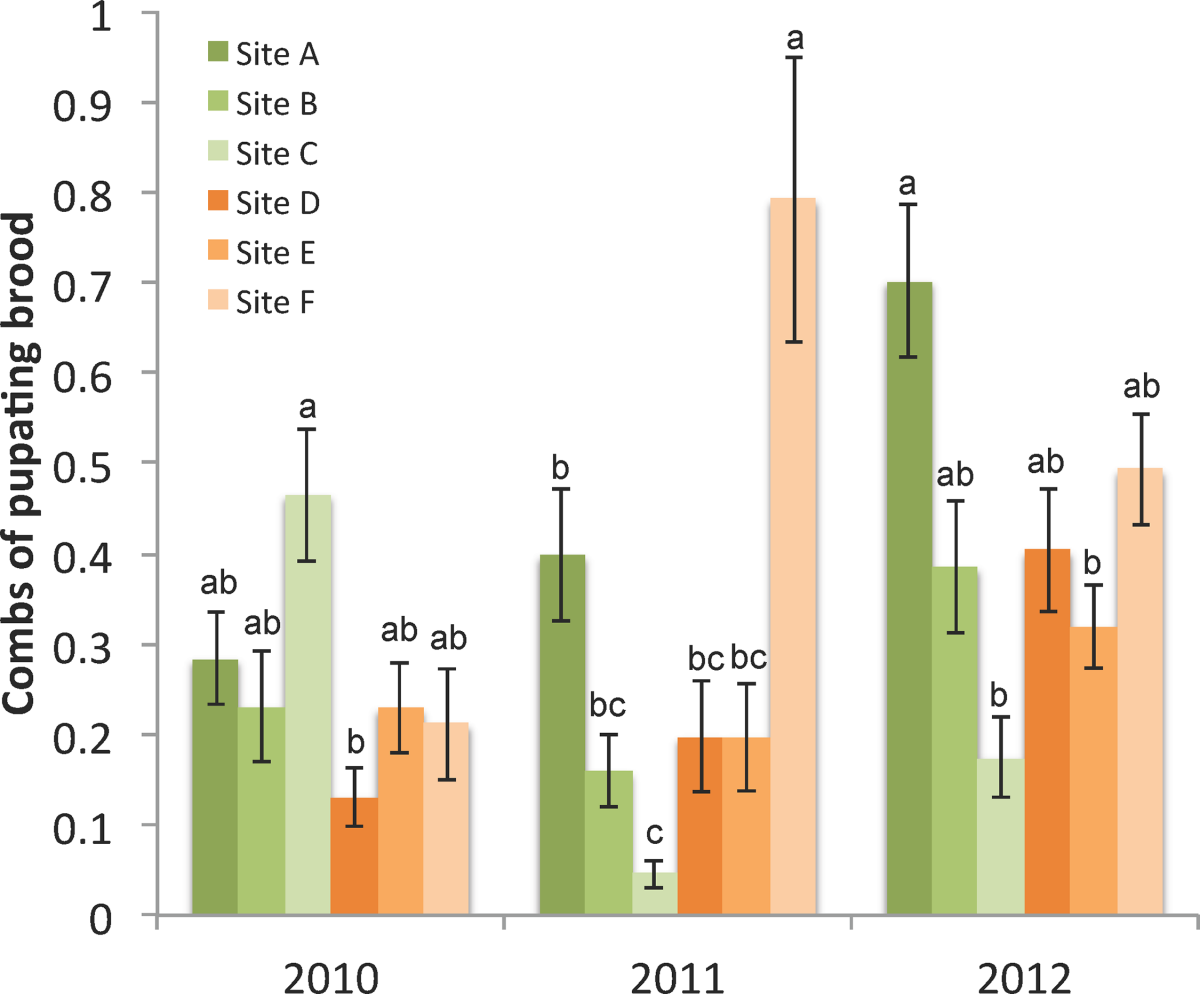
Pupating brood population. The mean area (summed number of combs) containing pupae ± s.e. by site and year, n = 24 colonies per site). Letters denote sites with significantly different total areas of pupating brood within each year.

As with adult population, the amount of brood in colonies varied widely throughout the year. Maximum brood area, measured as the total area (converted to number of combs) containing pupae occurred in July-September of each year. Minimum brood area occurred from September through January, when little brood rearing generally occurs in honey bee colonies. Across the years, there were significant interactions between site and date in the amount of pupating brood in colonies (F_95,2561_ = 1.82, p<0.0001). The amount of brood in September of each year differed, interacting by site and year (S2 and S3 Tables) and there were differences among sites for each year (Fig 1).

#### Colony pollen collection and storage

**Fig 2:**
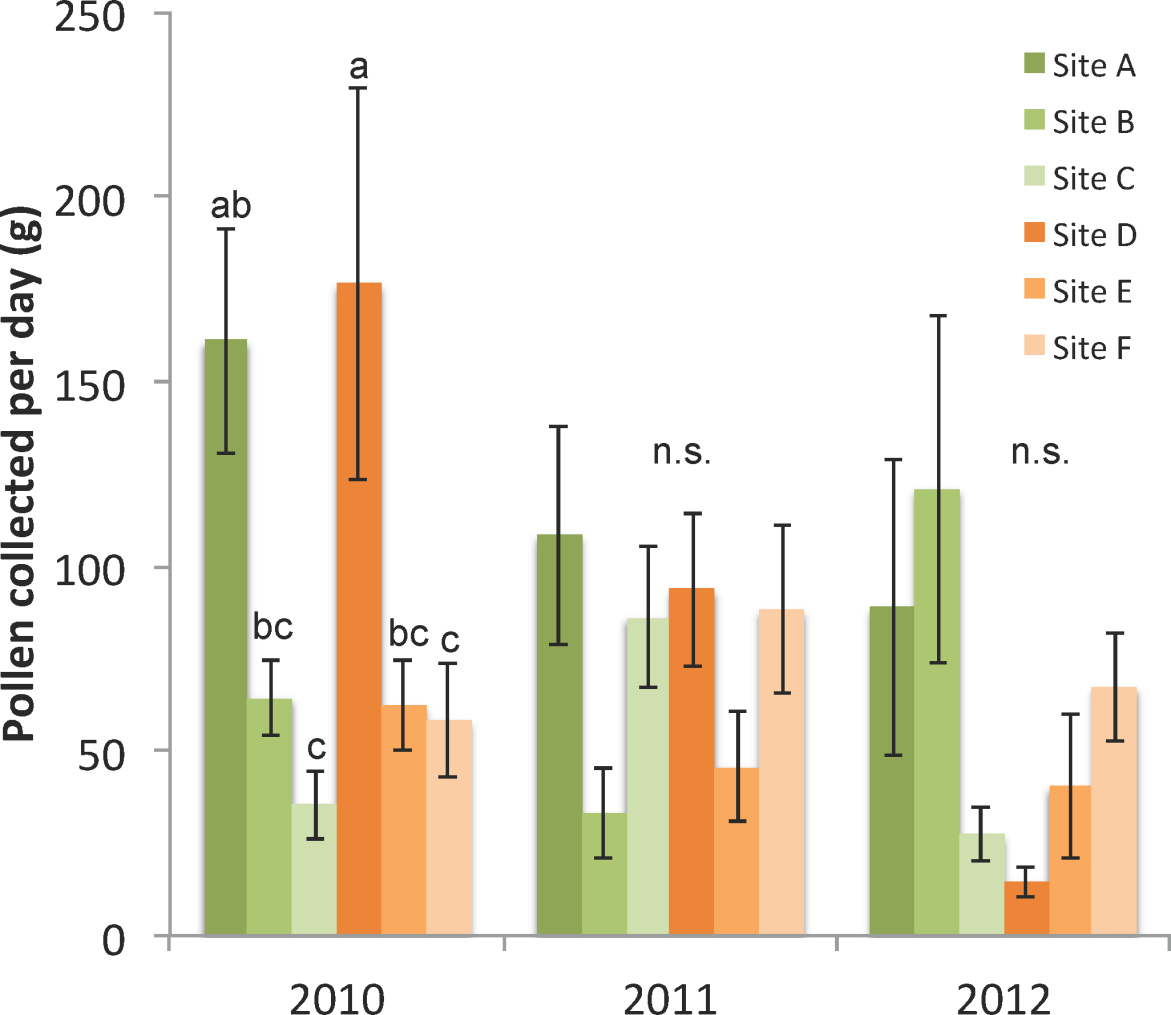
Pollen collection per day in each site and year, 2010–2012. Traps were open for 24 hours and fresh weight (g) of incoming pollen from three colonies per site were averaged on each date (± s.e.). Letters denote significant differences among sites within each year.

Pollen collection, the fresh weight of incoming pollen collected in traps per 24-hour sampling period, was measured 1–2 times per month in 3 colonies per site over the summer (up to 6 samples in 2010, 5 in 2011, and 3 in 2012). There was a high degree of variance in pollen collection among the three colonies within a site and among years (Fig 2), leading to a significant interaction between site and year (S2 and S3 Tables), though a significant difference among sites occurred only in 2010 (Fig 2).

Pollen stored within the combs of colonies was measured in September. Among all years, site A generally had more pollen stored in combs by the end of the foraging season, though other sites also had large stores in particular years (S2 Fig). There was a significant interaction between site and year (F_10,392_ = 3.56, p<0.0001) for pollen stores. Within a year, there were only differences in pollen stores in 2011 (S2 Fig)

#### Queens and colony losses

**Fig 3:**
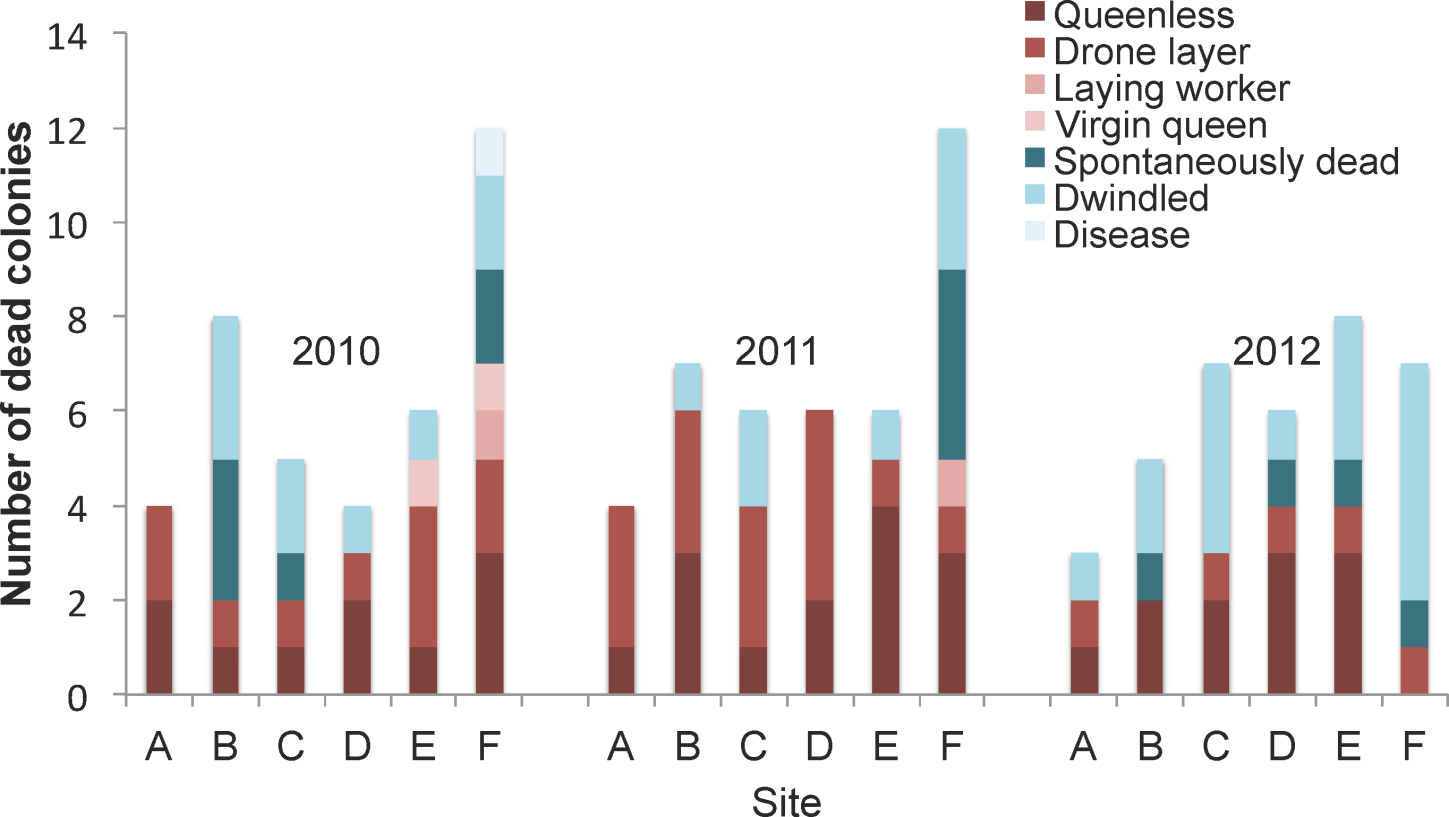
Status of colonies prior to mortality 2010–2013. Each apiary started out with a total of 24 colonies in North Dakota each May. Final colony losses were determined in March (almond pollination) of the following year. Status of the queen was assessed every 6 weeks in each colony.

New queens were introduced into each colony in the spring of each year and monitored during the life span of a colony, extending from May of one year (when colonies were in North Dakota) to March of the following year (when colonies were in almond orchards in California) from 2010–2013. Fig 3 shows the status of queens in colonies experiencing mortality in the colony assessment period six weeks prior to being reported as dead. Among all colonies (24 colonies in six sites over three years) the average annual colony mortality rate was 26%. Among the colonies that died (Fig 3), a majority (59%) had an apparent queen issue on the previous inspection (the colony was queenless, the queen was a drone layer, there was no queen but workers were laying eggs, or there was a virgin queen), while 28% dwindled over time with no signs of queen problems, and 12% perished absent an obvious diagnostic cause (no problems were evident during the previous assessment period). Only one colony death was potentially associated with an obvious disease, *Ascophera apis* in 2010 at site F.

#### Parasites and pathogens

**Fig 4:**
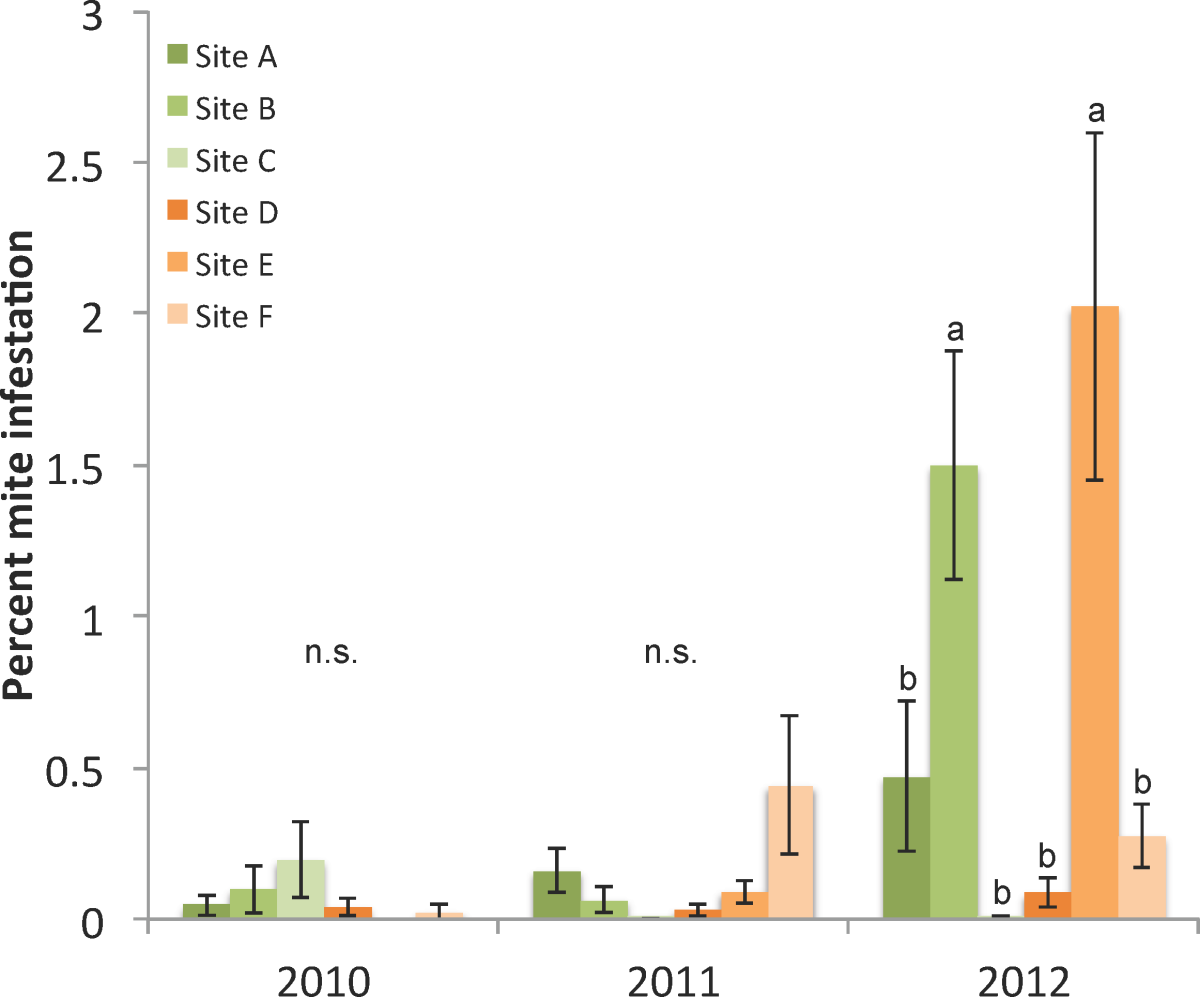
Levels of *Varroa destructor* in September shown as percent mite infestation per 100 adult bees. N = 24 colonies per site, letters denote significant differences among sites within each year.

*Varroa* mites were controlled by the beekeeper using the miticide amitraz twice per year. No apiary site consistently had colonies with the lowest or highest *Varroa* mite levels over time; i.e. there was an interaction in mite levels between site and date (S3 Fig). Infestation rate (mean number mites per 100 adult bees) generally increased over the course of the summer after treatment each May. In 2010 and 2011, mean mite levels never exceeded 1 mite/100 bees, while in 2012, mite levels were slightly higher but remained under 3 mites/100 bees. For the month of September (the time point for statistical modeling) an interaction occurred between site and year (Fig 4: F_10,393_ = 7.01, p<0.0001). Autumn mite treatment, coupled with the seasonal decline in colony brood-rearing, led to decreased *Varroa* infestation levels through January. By March, *Varroa* levels had increased before treatments were applied in May of each year (S3 Fig).

For *Nosema* spp. spore levels, there was a significant interaction between site and date (S4 Fig: F_85,2374_ = 3.5, p<0.0001). Peak *Nosema* infection levels occurred across at all sites from May-August, and minimum levels occurred from November-March, possibly in response to Fumagilin-b® treatments given to all colonies by the beekeeper in September and February each year. In September, there also was an interaction between site and year (S2 and S3 Tables) due to colonies in site C in 2011 having higher counts of *Nosema* spp. spores compared to all other sites.

**Fig 5:**
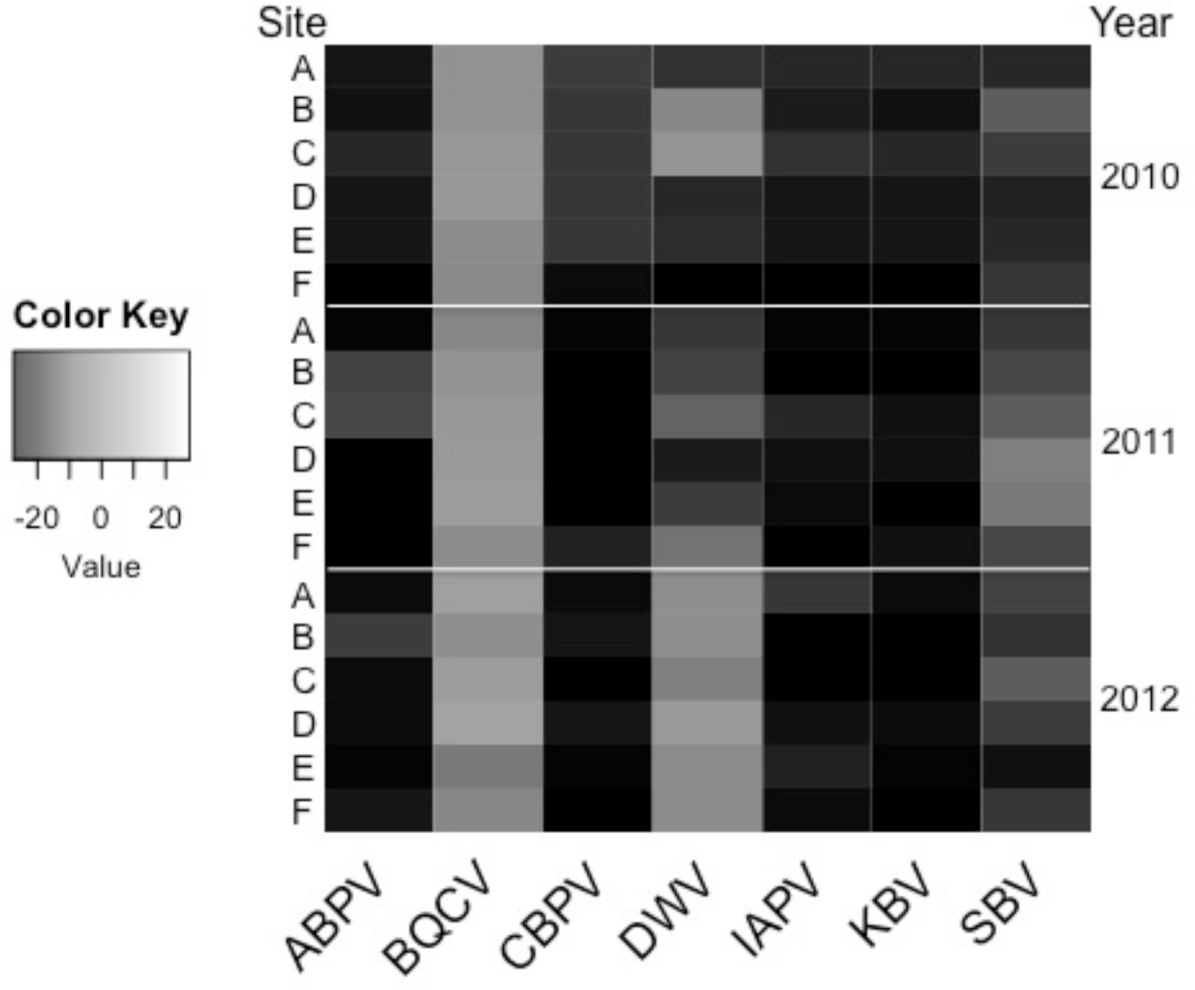
September viral expression by site and year, 2010–2012. Adult bees were taken from the brood chamber from each of the 24 assessed colonies at each site. Expression levels of viruses were determined relative to the reference gene, RPS5. The lowest levels of viral expression are depicted in black, while the highest levels are in white.

Viral titers in September varied over the course of the study (Fig 5) and generally followed one of three patterns: consistently low, consistently high, or variable among sites and years. Certain viruses including Acute bee paralysis virus (ABPV), Chronic bee paralysis virus (CBPV), Israeli acute paralysis virus (IAPV), and Kashmir bee virus (KBV) occurred at relatively low levels in all sites and years. Black queen cell virus (BQCV) was present at relatively higher levels in worker bees in colonies from all sites and years. Deformed wing virus (DWV) and Sacbrood virus (SBV) varied by site and year. Significant interaction between site and year occurred for most of the viruses measured in September, including ABPV, BQCV, DWV, IAPV, and SBV (S2 Table).

### Individual bee measures

#### Statistically modeled individual bee measures

**Fig 6:**
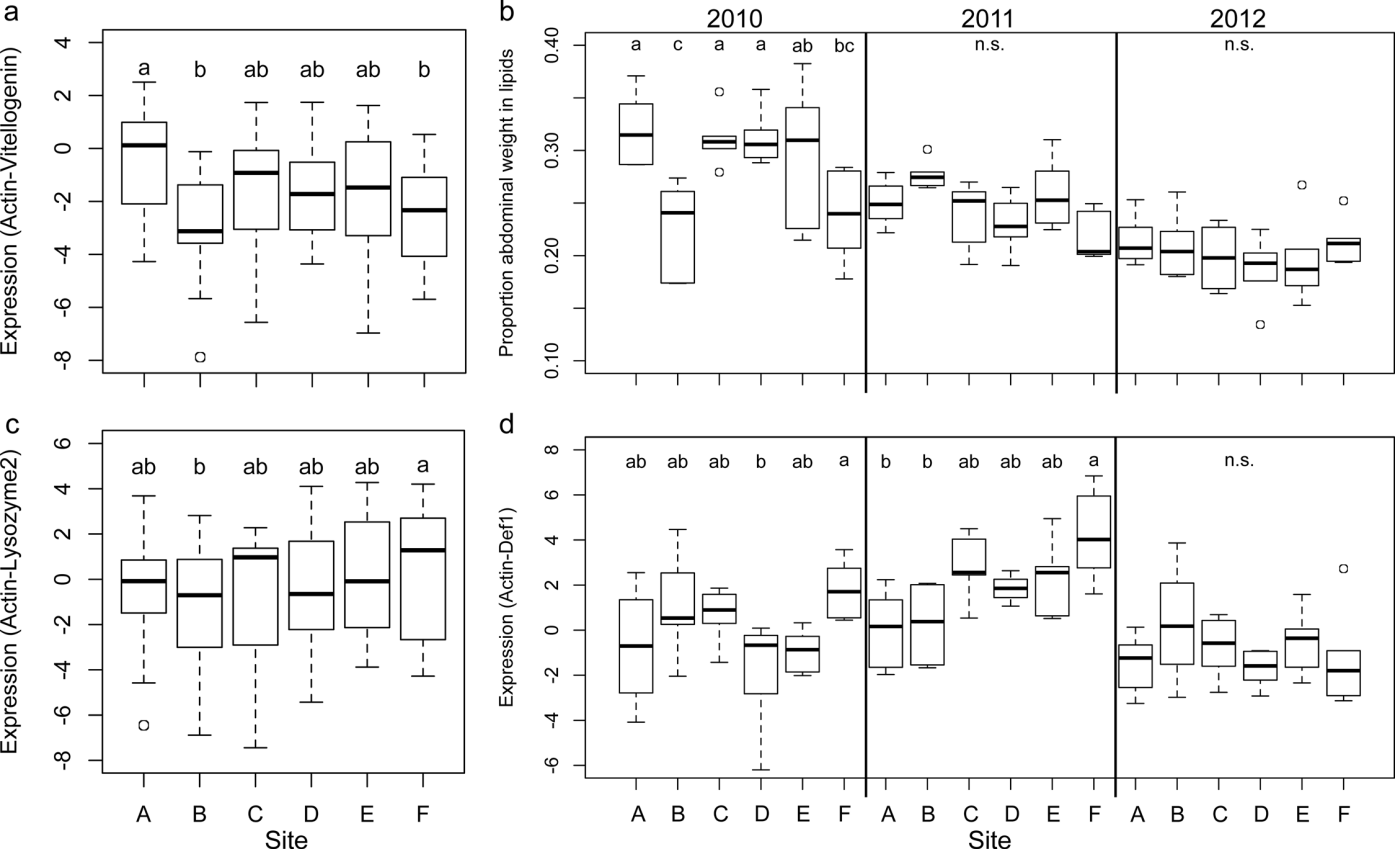
Measures of statistically modeled nutritional status and immune gene expression in individual 7-day old bees collected from colonies in September each year. Measures of nutrition included gene expression levels of *vitellogenin* (a) and weight of abdominal lipids (b). Measures of immune functioning included gene expression levels of *lysozyme2* (c), and *defenisin1* (d). (See S5 and S6 Figs for other related measures). *Vitellogenin*, *defensin1*, and *lysozyme2* expression were determined from the same bees (n = 47 per site), while abdominal lipids were quantified from separate bees (n = 180 per site). Box and whisker plots depict the median (black line), upper and lower 25% quartiles (the box), maximum and minimum (whiskers) not including outliers, and outliers (open circles) that were greater or less than 1.5 times the upper or lower quartile, respectively.

Expression levels of all genes (nutrition and immune genes) were analyzed in 7-day-old adult bees each September as an indicator of health at the end of season (see S2 Table for test statistics). The expression levels were measured in a sample of bees from 6 colonies within each site and were used for modeling overwinter apiary survival. *Vitellogenin* expression varied significantly among sites (F_5,100_ = 4.26, p = 0.001) with site A exhibiting the highest expression levels of *vitellogenin* in all years (Fig 6a). Overall expression levels of *vitellogenin* were lower from bees in all sites in 2012 compared to the first two years of the study.

Abdominal lipid weights in bees collected in September varied across years, being highest in 2010 and lowest in 2012 (Fig 6B). An interaction occurred (F_10,90_ = 4.70, p<0.0001) between site and year, but differences among sites within a year only occurred in 2010 (Fig 6B). Lipid levels remained relatively constant over time, but were typically greatest in July (concurrently with greatest hypopharyngeal gland size and brood area) and January (S5 Fig). The lowest lipid levels generally occurred in August or September.

*Lysozyme2* expression levels in 7-day old bees varied significantly among sites (F_5,100_ = 2.81, p = 0.02) and years (F_2,100_ = 182.45, p<0.0001), but there were no interactions between the two. The highest levels occurred in bees from site F (Fig 6C). For *defensin1*, interactions between site and year were detected (F_10,90_ = 2.73, p = 0.006) and bees from site F experienced the highest expression of *defensin1* (Fig 6D). Bees from site A generally expressed the lowest levels of *defensin1*, however this varied by year.

#### Other individual bee measures

The expression of *insulin-like peptide1* (S6 Fig) was very similar to that of *vitellogenin*. Site A expressed generally higher levels of *insulin-like peptide1* in all years. Hypopharyngeal gland size of bees collected in September varied across sites and years (interaction term: F_10,90_ = 7.38, p<0.0001) (S6 Fig). Overall, gland sizes were largest in 2010 and smallest in 2012. Peak hypopharyngeal gland size occurred in July (S5 Fig) concurrently with peak lipid levels and brood area. Gland sizes increased again the following March when the bees were positioned in CA almond orchards where they were actively collecting pollen and beginning to increase in population size and brood rearing. Minimum average size of glands occurred in January, a time when there was very little brood requiring food from the glands. For the remaining two immunity related gene expression measures (*abaecin*, *hymenoptaecin*) site A generally had low levels, while site F had the highest levels (S6 Fig, statistics in S2 and S3 Tables). Expression levels of all immune genes were generally opposite to that of the nutritionally-related genes, particularly *vitellogenin*.

### Statistical models of colony and individual bee measures on survival

The best statistical colony predictors of apiary survival, based on linear mixed effect modeling, were the mean grams of pollen collected per day, pupal brood area in September, and *Varroa* mite infestation levels in September (Table 3). The amount of pupating brood in September and amount of pollen collected over the season had positive influences on colony survival, while higher *Varroa* infestation levels in September were associated with decreased survival. Examination of the evidence ratios of the best models of survival indicated that the model that included all three colony measures had approximately 2 times the support compared to the model that included only mean pollen and September brood (*E* = 0.642/0.286), and approximately 13 times more support than the model including only September brood area (*E* = 0.642/0.051). The 95% confidence intervals for the top three model coefficients did not overlap zero (Table 3), further indicating that the combination of pollen collection, September brood, and September *Varroa* mites were the best colony level indicators of apiary survival.

**Table 3:** Colony level linear mixed effect models relative to annual number of colonies surviving among six apiaries in North Dakota, 2010–2013.

Response	Model	K	AICc	ΔAICc	*w*	Coefficients ± 95% C.I.
Number of surviving colonies per apiary	Pollen+brood+*Varroa*	7	79.87	0	0.642	Intercept: 13.86
						Pollen: 0.02 (0.01, 0.03)
						Brood: 7.05 (4.71, 9.14)
						*Varroa*: -1.12 (-1.85, -0.27)
	Pollen+brood	6	81.49	1.62	0.286	Intercept: 14.22
						Pollen: 0.02 (0.01, 0.03)
						Brood: 5.71 (3.12, 8.46)
	Brood	5	84.95	5.08	0.051	Intercept: 16.17
						Brood: 4.45 (1.24, 8.09)
	Brood+*Varroa*	6	88.33	8.46	0.009	Intercept: 16.19
						Brood: 5.05 (1.59, 8.85)
						*Varroa*: -0.68 (-1.96, 0.64)
	Pollen	5	89	9.13	0.007	Intercept: 16.61
						Pollen: 0.01 (-0.01, 0.03)
	Adult bees	5	90.81	10.94	0.003	Intercept: 15.88
						Adult bees: 0.15 (-0.75, 0.94)
	*Varroa*	5	90.93	11.06	0.003	Intercept: 17.64
						*Varroa*: -0.10 (-1.52, 1.27)
	Pollen+*Varroa*	6	93.57	13.7	0.001	Intercept: 16.65
						Pollen: 0.01 (-0.004, 0.03)
						*Varroa*: -0.19 (-1.63, 1.32)

K represents the number of parameters; ΔAICc represents the difference between AICc values of each model and the top-ranking model; *w* is the AICc model weight. Models are arranged from greatest evidentiary support (lowest AICc, highest *w*) to least support (highest AICc, lowest *w*). Pollen is the mean grams of pollen collected in pollen traps per 24-hr sample date; Brood is the mean September colony brood (pupae) area; *Varroa* is the mean September number of mites per 100 adult bees; Adult bees is the mean number of combs covered with adult bees in September.

Individual bee metrics that best indicated survival included a nutritional measure, the glycolipoprotein vitellogenin, and two immunity measures (*defensin1* and *lysozyme2* expression). The best model included expression of the immune gene encoding the anti-microbial peptide defensin1 alone: the higher the expression of this immune gene transcript, the lower the colony survival. The next best models included the expression levels of *vitellogenin* and immune peptide *lysozyme2* (second best model), and *vitellogenin* plus *defensin1* (third best model). Higher expression levels of *defensin1* and *lysozyme2* were associated with decreased survival, while higher levels of *vitellogenin* were related to improved overwinter survival. Evidence ratios indicated that the best model was 2 times more supported than the second best model (0.383/0.165) and 4 times more supported than the third best model (0.383/0.100) in which the 95% confidence intervals for the *vitellogenin* coefficient overlapped zero (Table 4).

**Table 4:** Individual bee level linear mixed effect models relative to annual number of colonies surviving among six apiaries in North Dakota, 2010–2013.

Response	Model	K	AICc	ΔAICc	*w*	Coefficients ± 95% C.I.
Number of surviving colonies per apiary	*def1*	5	86.28	0	0.383	Intercept: 17.78
						*def1*: -0.50 (-0.97, -0.01)
	*vg*+*lys2*	6	87.96	1.68	0.165	Intercept: 20.04
						*vg*: 1.61 (0.72, 2.44)
						*lys2*: -1.03 (-1.59, -0.47)
	*vg*+*def1*	6	88.97	2.69	0.1	Intercept: 18.64
						*vg*: 0.43 (-0.09, 1.02)
						*def1*: -0.74 (-1.24, -0.23)
	Lipids+*def1*	6	89.18	2.9	0.09	Intercept: 15.17
						Lipids: 10.68 (-6.55, 27.41)
						*def1*: -0.51 (-0.95, -0.06)
	Lipids	5	89.28	3	0.085	Intercept: 11.55
						Lipids: 24.80 (-0.10, 49.07)
	*lys2*	5	89.55	3.27	0.075	Intercept: 17.54
						*lys2*: -0.18 (-0.48, 0.10)
	Lipids+*lys2*	6	89.68	3.4	0.07	Intercept: 12.53
						Lipids: 20.16 (0.10, 39.59)
						*lys2*: -0.35 (-0.70, -0.05)
	Lipids+*vg*+*lys2*	7	92.86	6.58	0.014	Intercept: 17.02
						Lipids: 10.26 (-13.52, 37.30)
						*vg*: 1.30 (0.22, 2.42)
						*lys2*: -0.96 (-1.52, -0.43)
	*vg*+*def1*+*lys2*	7	93.18	6.9	0.012	Intercept: 19.79
						*vg*: 1.34 (0.03, 2.67)
						*def1*: -0.29 (-1.37, 0.88)
						*lys2*: -0.75 (-1.99, 0.54)
	Lipids+*vg*+*def1*	7	94.43	8.15	0.007	Intercept: 17.23
						Lipids: 4.63 (-22.12, 28.76)
						*vg*: 0.29 (-0.47, 1.17)
						*def1*: -0.67 (-1.25, -0.15)
	Lipids+hpg+*vg* +*def1*+*lys2*	9	108.09	21.81	<0.001	Intercept: 16.80
						Lipids: 8.43 (-21.74, 38.59)
						Hpg: 5.40 (-88.19, 107.85)
						*vg*: 1.27 (-0.01, 2.54)
						*def1*: -0.14 (-1.24, 0.93)
						*lys2*: -0.87 (-2.08, 0.23)

K represents the number of parameters; ΔAICc represents the difference between AICc values of each model and the top-ranking model; *w* is the AICc model weight. Models are arranged from greatest evidentiary support (lowest AICc, highest *w*) to least support (highest AICc, lowest *w*). *Vg*, *def1*, and *lys2* are mean gene expression levels relative to *β-actin* in September nurse bees. Lipids are the mean proportion abdominal lipid weight and hpg is the mean size (μm) hypopharyngeal gland acini in September nurse bees.

### Combined model of colony and individual bee measures

The measures that were best related to apiary survival from the colony and individual bee models were then brought together into a single combined model (Table 5). One potential problem arising from combining the two levels of data is exaggeration of weights of models containing related measures. We did not observe strong correlative relationships among any of the combined model measures. The first combined model included all three colony measures (pollen collection over the summer, brood and *Varroa* levels in September) and the expression of the gene encoding the anti-microbial peptide defensin1 in September. Subsequent models included the expression of the genes encoding for the glycolipoprotein vitellogenin and immune peptide lysozyme2. Higher levels of *Varroa* infestation, and higher expression levels of *defensin1* and *lysozyme2* were associated with decreased survival, while more pollen collection over the season, larger brood areas in September, and higher levels of *vitellogenin* in September, were related to improved overwinter survival. In all models, including the model with the greatest weight, individual bee measure confidence intervals overlapped zero, suggesting that the colony measure were more predictive of survival than any of the individual bee measures.

**Table 5:** Combined colony and individual bee level linear mixed effect models relative to annual number of colonies surviving among six apiaries in North Dakota, 2010–2013.

Response	Model	K	AICc	ΔAICc	*w*	Coefficients ± 95% C.I.
Number of surviving colonies per apiary	Pollen+brood+*Varroa*+*def1*	8	83.23	0	0.282	Intercept: 14.18
						Pollen: 0.02 (0.02, 0.03)
						Brood: 6.22 (4.14, 8.55)
						*Varroa*: -1.10 (-1.78, -0.44)
						*def1*: -0.21 (-0.43, 0.03)
	Pollen+brood+*def1*	7	84.55	1.32	0.146	Intercept: 14.61
						Pollen: 0.02 (0.01, 0.03)
						Brood: 4.74 (1.94, 7.51)
						*def1*: -0.25 (-0.56, 0.08)
	Pollen+brood+*Varroa*+*lys2*	8	85.11	1.88	0.11	Intercept: 13.99
						Pollen/day: 0.02 (0.02, 0.03)
						Brood: 6.52 (4.40, 8.76)
						*Varroa*: -1.31 (-2.10, -0.64)
						*lys2*: -0.10 (-0.29, 0.05)
	Pollen+brood+*vg*	7	85.59	2.36	0.087	Intercept: 14.38
						Pollen: 0.02 (0.01, 0.03)
						Brood: 6.49 (3.83, 9.14)
						*vg*: 0.22 (-0.20, 0.58)
	Pollen+brood+*def1*+*vg*	8	86.28	3.05	0.061	Intercept: 15.09
						Pollen: 0.02 (0.01, 0.03)
						Brood: 5.62 (3.12, 8.06)
						*def1*: -0.38 (-0.68, -0.04)
						*vg*: 0.39 (0.05, 0.73)
	Pollen+brood+*Varroa*+*vg*	8	86.67	3.44	0.051	Intercept: 13.87
						Pollen: 0.02 (0.01, 0.04)
						Brood: 7.07 (4.96, 9.35)
Number of surviving colonies per apiary						*Varroa*: -1.11 (-1.93, -0.21)
						*vg*: 0.01 (-0.25, 0.37)
	Pollen+brood+*lys2*	7	87.04	3.81	0.042	Intercept: 14.20
						Pollen: 0.02 (0.01, 0.03)
						Brood: 5.76 (2.72, 8.64)
						*lys2*: 0.01 (-0.22, 0.22)
	Pollen+brood+*def1*+*lys2*	8	87.05	3.82	0.042	Intercept: 14.56
						Pollen: 0.02 (0.01, 0.03)
						Brood: 5.62 (3.29, 7.99)
						*def1*: -0.56 (-0.96, -0.19)
						*lys2*: 0.29 (0.03, 0.58)
	Pollen+*def1*	6	87.17	3.94	0.039	Intercept: 16.58
						Pollen: 0.02 (-0.001, 0.03)
						*def1*: -0.54 (-0.99, -0.08)
	Brood+*def1*	6	87.94	4.71	0.027	Intercept: 16.62
						Brood: 3.36 (-0.46, 6.94)
						*def1*: -0.28 (-0.72, 0.20)
	Pollen+brood+*lys2*+*vg*	8	88.11	4.88	0.025	Intercept: 15.56
						Pollen: 0.02 (0.01, 0.03)
						Brood: 5.66 (2.85, 8.15)
						*lys2*: -0.41 (-0.78, 0.05)
						*vg*: 0.81 (0.14, 1.47)
	Brood+*vg*	6	88.3	5.07	0.022	Intercept: 16.35
						Brood: 5.43 (1.64, 9.30)
						*vg*: 0.28 (-0.20, 0.77)
	Pollen+brood+*Varroa*+*lys2*+*vg*	9	88.7	5.47	0.018	Intercept: 14.86
						Pollen: 0.02 (0.02, 0.03)
						Brood: 6.46 (4.48, 8.33)
						*Varroa*: -1.14 (-1.78, -0.48)
Number of surviving colonies per apiary						*lys2*: -0.36 (-0.65, -0.10)
						*vg*: 0.54 (0.04, 1.02)
	Brood+*lys2*	6	89.59	6.36	0.012	Intercept: 16.15
						Brood: 4.52 (0.80, 8.32)
						*lys2*: 0.01 (-0.29, 0.29)
	Pollen+brood+*Varroa*+*def1*+*vg*	9	90.34	7.11	0.008	Intercept: 14.48
						Pollen: 0.02 (0.02, 0.03)
						Brood: 6.36 (4.38, 8.45)
						*Varroa*: -0.87 (-1.63, -0.19)
						*def1*: -0.28 (-0.58, -0.03)
						*vg*: 0.18 (-0.13, 0.52)
	*Varroa*+*def1*	6	90.73	7.5	0.007	Intercept: 17.87
						*Varroa*: -0.28 (-1.74, 1.32)
						*def1*: -0.51 (-1.00, -0.10)
	Pollen+*lys2*	6	91.23	8	0.005	Intercept: 16.33
						Pollen: 0.02 (-0.002, 0.03)
						*lys2*: -0.22 (-0.48, 0.04)
	Pollen+*def1*+*vg*	7	91.45	8.22	0.005	Intercept: 17.27
						Pollen: 0.01 (-0.003, 0.03)
						*def1*: -0.69 (-1.14, -0.22)
						*vg*: 0.30 (-0.23, 0.86)
	Pollen+brood+*Varroa*+*def1*+*lys2*	9	91.46	8.23	0.005	Intercept: 14.22
						Pollen/day: 0.02 (0.01, 0.03)
						Brood: 6.25 (4.22, 8.57)
						*Varroa*: -0.95 (-1.70, -0.16)
						*def1*: -0.29 (-0.67, 0.18)
						*lys2*: 0.07 (-0.23, 0.35)
Number of surviving colonies per apiary	Pollen+*def1*+*lys2*	7	92.24	9.01	0.003	Intercept: 16.75
						Pollen: 0.02 (-0.001, 0.03)
						*def1*: -0.72 (-1.38, 0.03)
						*lys2*: 0.14 (-0.29, 0.59)
	Pollen+*vg*	6	93.48	10.25	0.002	Intercept: 16.37
						Pollen: 0.01 (-0.006, 0.03)
						*vg*: -0.11 (-0.62, 0.44)
	*Varroa*+*lys2*	6	93.49	10.26	0.002	Intercept: 17.71
						*Varroa*: -0.65 (-2.24, 0.96)
						*lys2*: -0.24 (-0.53, 0.11)
	*Varroa*+*vg*	6	95.51	12.28	0.001	Intercept: 17.54
						*Varroa*: -0.20 (-1.94, 1.56)
						*vg*: -0.07 (-0.67, 0.52)

K represents the number of parameters; ΔAICc represents the difference between AICc values of each model and the top-ranking model; *w* is the AICc model weight. Models are arranged from greatest evidentiary support (lowest AICc, highest *w*) to least support (highest AICc, lowest *w*). Pollen is the mean grams of pollen collected in pollen traps per 24-hr sample date; Brood is the mean September colony brood (pupae) area; *Varroa* is the mean September number of mites per 100 adult bees. *Vg*, *def1*, and *lys2* are mean gene expression levels relative to *β-actin* in September nurse bees. Lipids are the mean proportion abdominal lipid weight in September nurse bees.

## Discussion

### Colony health

This study identified significant measures of the health of honey bee colonies, and individual bees within those colonies, located in North Dakota during the summer months that predicted apiary survivorship after the colonies were moved to California to pollinate almonds the following spring. Of all the measures taken, three colony level metrics stood out as most strongly relating colony health to survival: 1) average amount of fresh pollen collected per day over the summer, 2) the total number of combs of sealed brood (pupae) in September, and 3) *Varroa* mite infestation levels in September. The remaining colony measures, including those related to colony size, disease occurrence and levels, and pesticide exposure among sites failed to impact survival in a statistically significant manner. Throughout this study, the beekeeper regularly and effectively controlled diseases and parasites, which may explain why pathogen levels were not related to survival. Also residue analysis of pesticides in pollen indicated that pesticide exposure may not have reached the levels of acute toxicity [29].

The amount of brood and level of *Varroa* mites in September were key parameters in the model. Colonies with larger brood areas in September tended to survive winter better while colonies with higher mite levels tended to have lower survivorship over the winter months. The average mite infestation per site, and the infestation rate for most colonies within a site, were below those known to cause harm to colonies (e.g. approximately 3 mites/100 bees in the fall) [30], showing that the beekeeper was adequately controlling mite populations. There was, however, enough variation, particularly in 2012, to maintain *Varroa* as an important predictive survival variable in our statistical models, and we would expect *Varroa* to be an even more influential factor in overwintering survival of colonies and sites with populations over acceptable thresholds [10].

Our results suggest that when mite infestations are low and honey stores are abundant, a large brood area in autumn may lead to more bees contributing to better thermoregulation in the winter cluster (though not necessarily to a greater number of long-lived winter bees) [31, 32]. At the same time, a larger winter population would potentially consume more resources, and greater areas of pupating brood in a colony in autumn could contribute to higher late season mite loads. Therefore beekeepers must balance adequate colony population size with amount of stored resources for successful overwintering. Our findings also emphasize the importance of monitoring and efficaciously managing *Varroa* mite populations in early fall to ensure winter survival. The amount of developing brood, measured as the total area of pupae in September, was predictive of colony survivorship the following spring. Sites with colonies containing larger brood areas in September tended to survive winter better. Commercial pollination of almonds in early spring necessitates a truncated winter for colonies, so a larger brood population going into a short winter may have been advantageous in our study.

Adult bee population was not predictive of colony survivorship. This measure may have been confounded by the time of day when the assessment was made, and whether the ambient temperatures caused bees to cluster within the hive boxes. For example, more bees generally can be found in a colony in the early morning (though they may be more clustered) compared to the late morning or afternoon when more bees are involved with foraging efforts in the field and may be more diffused throughout the colony.

#### Surrounding Land use related to bee health

Site A had the highest survivorship over all three years and was surrounded by greater floral abundance [29]. Floral abundance near such an apiary likely made it easier for bees to locate and exploit pollen resources. In contrast, honey bees from site F, the site with the lowest annual survivorship, may have had to travel farther and/or locate and exploit smaller patches of flowers, depleting greater stored colony resources in an effort to support their colony population growth requirements over the growing season. Thus, while colony populations did not differ significantly among sites on most of the sample dates during the summer, end of season honey production and overwintering colony survival were significantly impacted as a result of the varying spatial patterns of resource availability surrounding the sites [29].

One question that remains is how floral resources are allocated by colonies to improve their physiological health and survival, specifically in relation to pollen. Site A had more total area in bee forage [29] and higher amounts of pollen were brought into colonies at site A compared to the other sites. Despite the abundance of forage and realized amount of pollen collected, these resources apparently did not translate into observable differences in colony population size or amount of pollen stored in the colonies by fall, suggesting that the increased flower abundance available at site A likely was directly consumed by the bees rather than being allocated into colony storage. Thus, those protein resources must have been assimilated into the existing bee population and was manifested in our measurements of the improved nutritional status of bees at those sites.

The larger amount of bee forage at particular sites likely resulted in a decreased expenditure of individual bee energy (nutritional) stores and colony-level energy (nectar and honey) stores while bees located and collected pollen and nectar resources in the environment. Colonies experiencing an increased nutritional status (higher *vitellogenin*, *insulin-like peptide1*, and lipids) generally originated from sites with more potential forage (e.g. sites A, C, D). The colonies at these sites also tended to produce more honey on average. Also potentially related to a better quality landscape was the fact that only one colony from site A dwindled and no colonies at this site died absent apparent previous symptoms (“spontaneous death”) over the entire study period.

#### Queens

Queen problems were a common issue across all sites, despite annual beekeeper replacement of queens in all colonies. Queenless colonies and drone layers were common (8–50% across sites and years) immediately before colony death, however it should be noted that queen-right colonies also experienced death. Depending on when queen loss (in the case of queenlessness) or drone laying queens occurs, the beekeeper may salvage otherwise doomed colonies via queen replacement. In this study, approximately 40% of colonies experiencing a queen problem (which occurred in 17% of all colonies) showed signs of potential queen failure between May and September, meaning the majority (60%) of queen failure occurred over the winter with no prior colony signs suggestive of queen failure. Queen failure may occur for a variety of reasons including poor queen mating, low drone sperm quality, queen and/or drone pesticide and disease exposure, inappropriate beekeeper-mediated queen introduction, natural queen senescence, and other unknown factors involving a lack of colony acceptance of introduced queens [33–38]. Causes of queen failure are an active area of current honey bee research.

#### Parasites and pathogens

Parasites and pathogens are often linked to colony losses (e.g. [1,10,39]). We failed to detect any meaningful differences in the occurrence or levels of *Varroa* and *Nosema* levels among the sites, likely because the beekeeper maintained an effective management strategy to control both of them. Further, in the case of *Nosema*, spore counts and actual infection levels may not be well correlated [40]. Colonies were “homogenized” when transported to California and again when transported back to North Dakota, affording opportunities to transmit diseases and parasites among each other (e.g. during transport on trucks, in holding yards, in almond orchards). In this study there might not have been sufficient differences in pesticide use surrounding the study apiaries to detect interactions between pesticides and diseases potentially influenced by surrounding land use management (e.g. [8]).

Many of the known honey bee viruses are associated with *Varroa* mites but also seem to occur seasonally and/or ubiquitously [13] in bees without causing obvious deleterious effects. For reasons outlined above, it is perhaps not surprising that no large differences in virus levels were observed among the sites. At least one virus that we detected at relatively high levels at all sites, DWV, is highly associated with *Varroa* mites, and overwintering bees may be particularly susceptible to such viral infection [41]. Nutrition is known to interact with *Varroa* mites, viruses, and the immune system; thus bees and colonies may exhibit an increased susceptibility to certain diseases via poor nutrition or the presence of *Varroa* mites that reduce nutrient stores and suppress immunity [4,16,42].

### Individual bee health

Nutrition and immune measures in individual 7 day-old nurse bees collected from colonies in September revealed four metrics strongly relating bee health to survival: gene expression levels of 1) *defensin1* 2) *vitellogenin*, 3) *lysozyme2*, and to a lesser degree, 4) weight of abdominal lipids. Higher levels of *vitellogenin* and lipid weights were positively related to survival, while higher transcription levels of antimicrobial peptides *defensin1* and *lysozyme2* were negatively associated with apiary survival. Compared to the top colony model, the top statistical model of individual bee measures was not robustly supported; the weight of the most supported individual bee model was only 2–6 times that of the next 6 best models (though it had 26–50+ times the support of the remaining models). However, it should be noted that all of these (top 7) models contained some combination of *defensin1*, *vitellogenin*, *lysozyme2*, and abdominal lipids, suggesting some convergence on this particular set of individual bee metrics. These measures correspond with previous laboratory studies on physiological responses of honey bees to varying protein feeding regimes [4,5,27] in which both *vitellogenin* expression and lipid stores, as well as the immune response, were affected by nutrition. Colonies at site A (and *vice versa* at site F) were surrounded by the most land in potential bee forage, had the most incoming fresh pollen per day, had higher levels of *vitellogenin* and lower levels of *defensin1* and *lysozyme2*, and experienced the greatest survival which indicates a connection at the field level between landscape scale forage availability and individual bee nutrition and immunity.

Manipulation of nutritional status (e.g. amino acid supplementation) impacts honey bee proteins that are nutritionally related, such as vitellogenin and insulin-like peptides [22]. The protein vitellogenin in particular plays multiple roles in honey bee nutrition, immunity, stress resistance, behavioral development, ageing, and longevity [21,43–47]. Long-lived, stress-resilient adult bees emerging in the late fall accumulate and maintain relatively high hemolymph and fat body vitellogenin titers throughout the winter [21,44]. Protein and fat production, including vitellogenin, primarily occur in the insect fat body that exerts effects on both nutrition and immunity [48]. Further, the relative lipid mass of the insect fat body has been previously used as an indirect proxy for age and nutritional state [26,27,49], immunocompetence [27,50] and longevity [51,52]. Interactions between nutritional status and immunity have been observed in other insects [53–57] and, further, resource allocation alteration among molecular signaling pathways in response to immune stimulation has been shown to occur in *Drosophila* [58].

The activities of anti-microbial peptides are thought to be quite broad, killing many strains of bacteria and fungi while having relatively low toxicity to the host organism [59]. Defensin-1, produced primarily in the head, may be involved in defense during brood-rearing [60] acting by disruption of the permeability of bacterial membranes [61] that leads to a loss of cytoplasmic ATP and inhibition of respiration [62,63]. Hymenoptaecin is thought to increase the permeability of the outer and inner membrane of invading bacteria [61,63]. Additionally, stimulation of humoral immune pathways is thought to trigger the production of lysozymes (1–3) in honey bees [64]; important enzymes involved in responding to both bacteria and fungi, and possibly promoting the expression of other AMPs [64–66]. Lysozyme activity against bacteria is achieved via cleavage of peptidoglycan bonds in the cell wall [67]. Further, expression of *lysozyme2* has been shown to be upregulated in response to infection by the causative bacterial agent of American Foulbrood (AFB), *P*. *larvae*, and the fungus, *Ascophaera apis* (causative agent of chalkbrood), in honey bees [66]. Expression of *lysozyme2* was similar to that of the three other antimicrobial peptides examined in our study (*abaecin*, *defensin1*, and *hymenoptaecin*) in that expression levels were generally highest at the site with the least potential bee forage within a 3.2-km radius (site F), and lowest at the most bee-friendly forage site (site A). Nutritionally stressed bees could have had unapparent infections (lack of colony signs) or may have invested in constitutive responses instead of the energetically costly inducible (AMP) immunity. We did measure total hemocyte counts in 2012 only, which were found to be lowest in bees from site A and highest in bees from site F.

Importantly, the six colonies per site from which individual bee were collected were normal and healthy upon inspection, and none died over any of the winters; thus the measures represent healthy colony average levels of nutrition and immunity among the six sites. Had all colonies, including those with queen problems, higher parasite and disease prevalence, and a lack of nutritional stores, been included in the sampling, even greater variation in measures might have been observed. Future studies are planned that will measure the individual fates of a broader sample of colonies and apiaries over the winter to relate to their fall physiological measures.

## Conclusions

Taken together, our results indicate that colonies positioned in apiaries surrounded by the most potential forage collect more pollen and nectar resources over the summer. Such colonies also generally contain more brood and lower *Varroa* mite infestation rates in the fall, and have a higher overwintering success rate. Individual honey bees within those colonies possess a quantifiably better nutritional status by the end of the foraging season (September). Likely as a result of this higher quality nutritional state, bees in those colonies displayed a less activated immune system, as evidenced by their decreased humoral immune response. Conversely, colonies positioned at sites with the least area in potential bee forage expressed some of the lowest nutritional stores (*vitellogenin*, *insulin-like peptide1*, lipids generally) and highest levels of humoral immunity. Importantly, our combined model was consistent with the two separate models of colony and individual bee measures (i.e. the best combined model contained pollen collection, brood, *Varroa*, and *def1*). The combined modeling indicated that colony measures were more strongly indicative of apiary survival that those taken from individual bees. Individual bee measures were taken from a fewer number of hives than colony measures, and none of the individual-bee sampled colonies actually died over the winter. These factors likely contributed to this result. We would expect a greater divergence of individual bee physiological indicators to emerge if taken from bees with a more varied colony health background, such as visually weak or diseased autumn colonies.

Potential trade-offs between nutrition and immunity have been previously observed in honey bees in the lab, specifically between *vitellogenin* and *defensin1*, wherein immune stimulation resulted in increased AMP defense and a concurrent decrease in *vitellogenin* expression [68]. Further, stimulation of the Toll signaling pathway in *Drosophila* has been shown to lead to a decrease in nutrient stores [58]. In terms of our field study, the improved nutritional (higher) and immunological (quieter) states of bees in September at sites with higher survival were those surrounded by more forage throughout the summer and this may explain the differences observed in overwintering survival among the study sites.

Future research on honey bee colonies positioned across a broader variety of landscapes and beekeeping practices is planned to test how robust the predictors of colony and apiary survival are. Efforts to identify absolute levels of particular genes and other physiological measures relating to health and survival may prove fruitful in providing beekeepers and researchers alike with a reliable tool or metric to accurately and objectively assess the status of their hives. If beekeepers were able to obtain measures of gene expression, it would enable them to assess and identify particular sites where colonies are at an increased risk of failure (due to relatively lower nutritional and/or higher immune measures) and thus may need to be closely monitored and possibly treated for parasites and diseases, or nutritionally supplemented. Beekeepers may even choose to withhold treatments and supplements from such colonies if they are likely to perish over the winter regardless of interventions. Such a “blood test” for bees would be a breakthrough in the ongoing struggle to maintain healthy, live colonies of honey bees to meet the current demands for pollination, support a robust beekeeping industry, and ensure a safe and reliable food system.

This study uncovered measures of honey bee health and immunity relating to apiary-level phenomena, i.e. apiary survival, influenced by landscape suitability. While much interest exists regarding the effects of land use on honey bee (and other pollinator) health and survival, little data exist on which to build coherent real-world policies that will significantly improve the situation for honey bees and other pollinators. As such, this study is a novel first step toward identifying pertinent physiological responses that honey bees may have as a result of their positioning near varying landscape features in intensive agricultural environments.

## Materials and Methods

### General methods

A total of 144 colonies were assessed over the course of 3 years (May 2010-March 2013) for measures of colony robustness and health. There were 48 colonies positioned at each of six apiary sites, which is typical for a commercial apiary. Of the 48 colonies, half (24) were assessed for colony level measures, and six colonies per site were used for individual bee collections and measures. Sampling and assessments occurred approximately every six weeks throughout the year except for gene transcript analyses of individual bees, which occurred in September only. All colonies were uniquely numbered for identification.

During the spring through fall months, colonies were positioned in North Dakota apiaries surrounded by landscapes of varying agricultural land use patterns (Table 1, [29]). Sites were ranked according to the total uncultivated forage area surrounding the apiaries; that is, the amount of forage land beneficial to honey bee colonies declined from A-F. The land use surrounding each site varied (Table 1), resulting in different amounts of floral resources that were available locally to the colonies. This ground survey data coupled with the pollen collection data (Fig 2) indicate that the land use surrounding certain sites, for example site A, was much richer in flower abundance which led to greater total pollen availability but not necessarily pollen diversity, though much variation occurred within and among sites [29].

Colonies were moved to holding yards in California in October of each year to overwinter before being moved to pollinate almonds beginning in mid-February of each year. Colonies were moved back to North Dakota each May where colonies and queens were replaced. Assessment and sampling locales, then, were in North Dakota during the months of May, July, August, and September, in California holding yards in November and January, and in California almond groves in March. Permission was obtained from all private landowners to position hives in their respective apiaries.

A certain amount of colony mortality is expected to occur annually in any beekeeping operation. Thus, the cooperating beekeeper placed new replacement colonies into the position of dead colonies each year in May in North Dakota, returning the total number of colonies at each site to 48 (24 assessed) at the beginning of each forage season. In addition, every colony was re-queened annually in April-May by removing the old queen and introducing a newly mated queen to the colony. Thus, the colony year began with the introduction of the new queen prior to the May sampling date and ended after the completion of almond pollination contracts the following March. Colonies dying during the course of each year were removed from the experiment for the purposes of determining summary statistics of colony measures at each site.

Typical commercial beekeeping practices to control pests and diseases were conducted on every colony by the beekeeper. *Varroa destructor* mite populations were suppressed using the miticide amitraz each spring and fall (May and August-September). *Nosema* spp. were controlled using Fumagilin-b® (Medivet Pharmaceuticals Ltd.) delivered in 1:1 sugar syrup twice per year in September and February. Additionally, all colonies were provided with pollen substitute in the spring.

This research did not involve endangered or protected species.

### Colony population size

The bee population was recorded within each of the 24 colonies by estimating the total number of frames (both sides of a wax comb contained within a removable wooden frame) covered with bees. Bee population was the first measure taken after opening a colony. Briefly, the lid was removed, the observer visually inspected the combs in the top box from above and below, and then the combs in the bottom box from above to approximate the number of combs completely covered by bees. The initial estimate was confirmed as the observer worked through the colony, comb by comb, and was revised as necessary.

The amount of brood in the pupal stage (sealed with a wax cap) present in the colony was determined by placing a frame-sized grid composed of 32 squares over each side of every frame within each colony similar to Delaplane et al. (2013) [69]. The number of squares containing sealed worker pupae, but not drone pupae, on each frame was then summed across frames to determine the total number of squares in the entire colony. The total number of squares for the entire colony was divided by 64 (to account for coverage of both sides of each comb).

### Colony nutritional resources

All colonies were provided additional boxes for the storage of honey (i.e. honey supers) by the beekeeper as needed over the course of the summer. The collaborating beekeeper maintained an adequate number of honey supers for colonies to continue storing honey. The amount of honey produced by each colony per year was determined by weight (kg) when the supers were removed. In cases where a colony died during the summer, supers were weighed before removing the colony from the experiment.

Total pollen storage in each colony was approximated by overlaying a grid (as described above) on each frame, summing the number of grid cells containing pollen, and dividing by the total number of possible grid cells (64 per frame) [69]. This measurement was taken only in August and September of each year as an indicator of stores for subsequent overwintering survival and spring build-up success [70–75].

An assessment of pollen collection was taken by maintaining three sentinel colonies at each of the six locations for pollen collection; these colonies were not included in the 24 regularly assessed colonies. The three colonies per site were fitted with pollen traps that, when activated, forced returning foraging honey bees to walk through screens upon entering the hive [76]. Pollen became dislodged from the hind tibiae of the returning foraging bee as the bee passed through the screen and fell into a pollen collection drawer at the bottom of the trap. Collections were conducted 6 times in 2010, 5 times in 2011, and 3 times in 2012. Collected pollen was bagged in the field and shipped frozen using dry ice and stored at -20°C until analyzed at the USDA-ARS bee laboratory in Beltsville, MD. Total weight (g) of fresh pollen was determined from each sample date, colony, and site.

### Colony queen status

Queen status was determined for each colony at the time of colony assessment. Each colony was inspected for the physical presence of the queen. However, it was often not possible to observe the queen in a colony with a large population of adult bees or when the colony had a high level of activity. In such cases, the presence of eggs and young larvae were used as a proxy for the presence of a laying, normally functioning queen. Abnormalities in egg-laying patterns were also recorded when observed. Because queen status was recorded on each assessment date, it was possible to characterize the prior status and/or abnormalities that may have contributed to the loss of those colonies, if they failed to survive. For example, queen problems included colonies in which the queen was laying only unfertilized drone eggs (drone layer), or the old queen had died and the colonies either was not able to raise a new queen from existing larvae (queenless), or the workers had begun laying eggs (laying worker). In a few instances a new unmated queen was observed in the nest (virgin) but the colonies ultimately failed to survive.

Colonies that possessed laying queens but nonetheless died were grouped into one of three categories: 1) spontaneously dead, where on the previous sample date a given colony appeared normal, robust, and queenright, 2) dwindling, where a colony was observed to lag or regress in population size over time before ultimately dying, and 3) diseased, where obvious disease symptoms were observed previously. The latter category was only observed once, in which the colony in question presented with a heavy chalkbrood (*Ascophaera apis*) infection.

### Parasites and pathogens

Adult bees were assayed for the occurrence and infestation/infection rate of commonly occurring parasites and pathogens from the 24 assessed colonies at each site. Approximately 300 adult bees were collected from a single comb into bottles containing 70% ethanol. The bees were mechanically shaken for 30 min. to completely dislodge the mites from the adult bees. After shaking, the total number of mites and bees was determined and used to calculate the infestation rate per 100 bees.

*Nosema* spp. spores per bee were determined from the same bees used to quantify the level of *Varroa* mite infestation. One hundred bees were crushed and homogenized with 100 mL DI water in a plastic bag on a marble slab using a rolling pin. Ten μL of the resulting homogenate were pipetted into a hemacytometer and the number of spores per bee was determined using a standard procedure [77].

A separate sample of adult bees was taken from the brood chamber of each colony to detect the occurrence of viruses and immediately placed on dry ice in the field, shipped, and stored at -80°C for later analysis. Total RNA was extracted from a composite of 50 bees per sampled colony using Trizol reagent (Invitrogen, Carlsbad, CA) following the manufacturer's protocol. Contaminating DNA was removed using DNAse I in an 11 μL reaction containing 8 μL (1.5 μg) total RNA, 10 U DNAse I (Invitrogen) in appropriate buffer, 20 U RNAseout (Invitrogen), poly dT_12-18_, and 2 mM dNTP. The DNAse reaction was performed at 37°C for 1 hr. followed by 75°C for 10 min. First-stand cDNA was synthesized by using 100 U Superscript II Reverse Transcriptase (Invitrogen) and incubation at 42°C for 50 min. followed by 15 min. at 70°C. The cDNA was diluted 1:5 in nuclease free water (~100 ng/μl). Quantitative PCR was performed in a 20 μl reaction mixture consisting of 1X SsoAdvanced^™^ SYBR^®^ Green supermix (Bio-Rad), 0.2 μM of each primer, and 1μl (~100 ng) of cDNA template (see S4 Table for primer sequences). The reaction was carried out in 96-well plates using a Bio-Rad Icycler (Bio-Rad Crop., Hercules, CA.) programmed with the following temperature profile: 95°C for 30 sec. followed by 50 cycles of 95°C for 5 sec., 60°C for 30 sec., melt curve from 65–95°C in 0.5°C/5 sec. increments. The melt curve dissociation was analyzed to confirm each amplicon.

### Collection of nurse bees for individual bee measures

Six colonies per apiary were semi-randomly selected (see below) for analysis of individual bee nutrition and immunity as it was not feasible to analyze individual bees from every colony. Each of the sampled colonies was first assessed at the colony level to ensure that there were no queen issues, presence of diseases and parasites, or other obvious abnormalities. Therefore, the individual bee measures represent apparently healthy colonies at each site.

As honey bee physiology changes with adult age, it was critical to analyze age-matched cohorts of bees. To accomplish this goal, newly-eclosed adult bees (≤24 hrs. after eclosion) were paint-marked (Testor’s enamel paint markers) on the dorsal surface of the thorax. Approximately 75 bees were marked per colony. The same paint color was never used twice within an apiary to eliminate the collection of drifting bees among colonies. Bees were allowed to develop normally in their colony of origin for seven days. After seven days, 15 marked bees were recovered from each colony and placed in cages provisioned with queen candy (powdered sugar mixed with water) and water. Cages were transported back to the University of Minnesota bee research lab in St. Paul, MN.

### Hypopharyngeal glands

The heads from 10 of the 15 caged nurse bees from each colony and date were separated from the thorax using a razor blade. The paired hypopharyngeal glands from each head were removed and placed in a glass well plate containing 500μL PBS (Lonza, Walkersville, MD). Gland size was measured with an ocular micrometer at 400x magnification using a Leica DM100 compound microscope [78]. The widths of the first 5 acini from the end of each gland were measured and averaged to determine the mean acinus diameter.

### Abdominal lipids

The abdomens from the same 10 bees used to determine hypopharyngeal gland size were also used to determine the amount of lipids in the abdomen of each bee. Each abdomen was separated from its thorax using forceps, followed by the removal of the alimentary tract. Each abdomen was placed into a sterile 1.5mL microcentrifuge tube and incubated at 70°C for 24 hrs. After 24 hours, the dry weight of each abdomen was recorded. Next, 300 μL of 1:1 chloroform:methanol was pipetted into each tube, and lids were quickly closed. The dried abdomens were incubated at room temperature for 24 hours, after which, the chloroform:methanol was removed via a vacuum pump. Tubes were then placed back in the drying oven for 24 hours at 70°C. Finally, the abdomens were re-weighed and the change in weight was used to determine the percent composition of lipids in the abdomen (i.e. (Starting weight–Final weight/Starting weight) x100).

### Individual bee gene transcript analysis

Total RNA was extracted from the abdomens of the 5 remaining 7-day old bees collected from each colony in September during the three years of the study according to the procedure provided with TRIzol^®^ Reagent (Life Technologies, Carlsbad, CA) to provide an end of summer assessment of health. Contaminating DNA was degraded, and mRNA was selected for, in the presence of the extracted RNA by preparing a master mix containing 240 U DNase I, 120 μL of 10x DNase buffer, 960 U RNase out, 24 mmol dNTPs, 240 mmol poly(dT)_18_, and 120 μg poly(dT)_12-18_. An aliquot of 3.1 μL of this master mix was added to each well of a 96-well plate, followed by 8 μL of total RNA. A BioRad MyCycler™ thermocycler was then run at 37°C for 1 hour followed by 75°C for 10 min. Next, first-strand cDNA was synthesized by adding 3.9 μL of master mix containing 100 U Superscript II reverse transcriptase (Invitrogen), 200 μL of 0.1 M DTT, and 150 μL 5X first-strand buffer to each sample well and running the thermocycler at 42°C for 50 min followed by 15 min at 70°C.

Two μL of cDNA from the previous step were pipetted into each well of a new plate and served as the template to determine the expression of gene transcripts via qPCR. Additionally, 18 μL dH_2_O, 0.15 μL of 5U/μL Taq polymerase, 2.5 μL 10x buffer, 0.2 μL dNTPs, 1.5 μL MgCl_2_, 0.05 μL 10x SYBR Green (Applied Biosystems), and 0.5 μL each of forward and reverse primers for *vitellogenin*, *insulin-like peptide1*, *lysozyme2*, *abaecin*, *defensin1*, and *hymenoptaecin* were added to each well for a total reaction volume of 25 μL (see S4 Table for primer sequences). Real-time PCR reactions were run in a Biorad C1000™ Thermal Cycler using a thermal profile consisting of 95°C for 10 min., then 94°C for 20 seconds, followed by 40 cycles of a protocol consisting of 4 steps: 95°C for 20 seconds, 60°C for 30 seconds, 72°C for 1 minute, and 78°C for 20 seconds. Fluorescence measures were taken repeatedly at the 78°C step. The 40^th^ cycle was followed by a three-step melt-curve dissociation analysis to confirm amplification of the targeted gene of interest. The relative expression of each gene of interest was determined as the Cq level (number of cycles for exponential amplification) of the gene of interest from the Cq level of the reference gene, ß-Actin.

### Statistical Analysis

We used repeated measures ANOVA including every colony for each measure across all dates to determine if a given colony health variable significantly differed by site and date. Tukey’s mean comparison test was used when factors were considered significant at the α ≤ 0.05 level. This ANOVA acted as starting points to inform selection of particular variables and sample dates to include in statistical modeling. We also quantified correlative relationships among measures, and eliminated those that were highly related to others so as to avoid redundancy in model variables. Parameters that were biologically and statistically (from above) relevant to the question—which measures taken from the colony and individual bees predict overwintering survival of apiaries—were reduced to 18 data points (6 sites by 3 years for a given measure among all sites and years) and included in statistical modeling using R version 3.1.1 (R core team, 2014-07-10) and the lme4 package [79]. The factors site and year were included as random effects while all other colony level predictors were considered as fixed effects.

For statistical modeling, we focused on measures taken from colonies at the end of the forage season (September) as this is a critical time point for the beekeeper and for overwintering colonies in terms of requiring adequate nutritional stores (in colonies and bees) and minimizing disease and parasite levels (e.g. *Varroa* mites and viruses). Examined measures included adult population size, pupal brood area, pollen storage area, pollen collection (g/day), honey production, *Varroa* mite infestation levels, *Nosema* spp. spore infection levels, virus expression, abdominal lipid stores, hypopharyngeal gland size, and nutritional and immunological gene expression. We hypothesized that colony metrics would be indicative of improved health among apiaries (strongly influenced by land use as shown in [29]) and those colonies expressing improved health would lead to better overwinter survival at the apiary level (number of surviving colonies).

Model parameters from the colony and individual bee measures were modeled separately to create two models (colony and individual bee) and important factors in those separate models were then brought together into a final combined model linking the respective measures to overall apiary site survival. Akaikae’s Information Criterion corrected for small sample size (AICc) was used to rank the multiple competing models relating health metrics to survival. We calculated AICc model weights (*w*) and evaluated coefficients and 95% confidence intervals to determine the relative importance of model parameters.

## Supporting Information

S1 Table Annual honey production, 2010–2012. Honey production is the mean kg honey per colony at each site.
(DOCX)

S2 Table ANOVA results for all colony and individual bee measures, 2010–2012. Results are for measures quantified in September unless otherwise indicated.
(DOCX)

S3 Table Tukey HSD comparisons of site x year interactions. Comparisons shown are those that were significant at p < 0.05 within a given date.
(DOCX)

S4 Table Primer sequences used for gene expression analysis. Colony level expression: viruses and reference gene, RPS5; Individual bee level expression: nutritional and immune genes and reference gene, ß-Actin.
(DOCX)

S1 Fig Colony adult population size, 2010–2013. Asterisks denote significant differences among sites on a given sample date.
(TIFF)

S2 Fig In-hive pollen storage, September 2010–2012. Letters denote significant differences among sites within each year.
(TIFF)

S3 Fig *Varroa* mite infestation levels, 2010–2013. Treatment with miticide occurred each September. Asterisks denote significant differences among sites on a given sample date.
(TIFF)

S4 Fig *Nosema* spore levels, 2010–2013. Treatment with Fumagillin was done each February and September. Asterisks denote significant differences among sites on a given sample date.
(TIFF)

S5 Fig Hypopharyngeal gland size and lipid stores for 7-day old nurse bees by site (mean ± s.e.), 2010–2013. Asterisks denote significant differences among sites on a given sample date.
(TIFF)

S6 Fig Other nutritional and immune measures. Individual bee measures quantified but not found to be informative for statistical modeling included the nutritional measures *insulin-like peptide1* (a) and the size of the hypopharyngeal glands (b). Immune-related measures included gene expression of *hymenoptaecin* (c) and *abaecin* (d).
(TIFF)

S1 Data Minimum data used for statistical modeling analyses. (CSV)
