# Supplementary material for: Linking Measures of Colony and Individual Honey Bee Health to Survival among Apiaries Exposed to Varying Agricultural Land Use
Source: PLoS One. 2016 Mar 30;11(3):e0152685. doi: 10.1371/journal.pone.0152685 (PMC4814072; doi:10.1371/journal.pone.0152685)
Supplement: S1 Table — Honey production is the mean kg honey per colony at each site. (DOCX) [file pone.0152685.s001.docx]

**S1 Table. Annual honey production, 2010-2012.** Honey production is the mean kg honey per colony at each site.

| Year | Site | Honey (kg) |  | S.D. |
| --- | --- | --- | --- | --- |
| 2010 | A | 47 |  | 14 |
|  | B | 27 |  | 14 |
|  | C | 46 |  | 17 |
|  | D | 30 |  | 11 |
|  | E | 34 |  | 15 |
|  | F | 12 |  | 12 |
| 2011 | A | 29 |  | 20 |
|  | B | 18 |  | 13 |
|  | C | 27 |  | 17 |
|  | D | 37 |  | 19 |
|  | E | 40 |  | 22 |
|  | F | 17 |  | 15 |
| 2012 | A | 64 |  | 28 |
|  | B | 50 |  | 21 |
|  | C | 52 |  | 24 |
|  | D | 36 |  | 16 |
|  | E | 34 |  | 18 |
|  | F | 44 |  | 24 |
