# Supplementary material for: Linking Measures of Colony and Individual Honey Bee Health to Survival among Apiaries Exposed to Varying Agricultural Land Use
Source: PLoS One. 2016 Mar 30;11(3):e0152685. doi: 10.1371/journal.pone.0152685 (PMC4814072; doi:10.1371/journal.pone.0152685)
Supplement: S2 Table — Results are for measures quantified in September unless otherwise indicated. (DOCX) [file pone.0152685.s002.docx]

**S2 Table. ANOVA results for all colony and individual bee measures, 2010-2012.** Results are for measures quantified in September unless otherwise indicated.

| **Measure** | **Effect** | **DF** | **Sum Sq** | **Mean Sq** | **F-value** | **P-value** |
| --- | --- | --- | --- | --- | --- | --- |
| Frames of bees | Site | 5 | 134 | 26.87 | 3.32 | 0.006 |
|  | Year | 2 | 9 | 4.45 | 0.55 | 0.58 |
|  | Site*Year | 10 | 310 | 31.02 | 3.84 | <0.0001 |
|  | Residuals | 392 | 3169 | 8.08 |  |  |
| Comb area containing pupating brood | Site | 5 | 5.1 | 1.02 | 10.09 | <0.0001 |
|  | Year | 2 | 1.76 | 0.88 | 8.73 | 0.0002 |
|  | Site*Year | 10 | 7.78 | 0.78 | 7.70 | <0.0001 |
|  | Residuals | 392 | 39.60 | 0.10 |  |  |
| Comb area containing stored pollen | Site | 5 | 9.23 | 1.85 | 5.79 | <0.0001 |
|  | Year | 2 | 10.12 | 5.06 | 15.86 | <0.0001 |
|  | Site*Year | 10 | 11.37 | 1.14 | 3.56 | 0.0002 |
|  | Residuals | 392 | 125.02 | 0.32 |  |  |
| Fresh weight (g) incoming pollen per summer | Site | 5 | 2.2x10^5^ | 4.4x10^4^ | 4.87 | 0.0003 |
|  | Year | 2 | 3.5x10^4^ | 1.7x10^4^ | 1.93 | 0.15 |
|  | Site*Year | 10 | 2.6x10^5^ | 2.6x10^4^ | 2.88 | 0.002 |
|  | Residuals | 234 | 2.1x10^6^ | 9.1x10^3^ |  |  |
| Honey per summer | Site | 5 | 1.0x10^5^ | 2.0x10^4^ | 12.2 | <0.0001 |
|  | Year | 2 | 1.3x10^5^ | 6.7x10^4^ | 40.3 | <0.0001 |
|  | Site*Year | 10 | 1.2x10^5^ | 1.2x10^4^ | 7.1 | <0.0001 |
|  | Residuals | 414 | 6.8x10^5^ | 1652 |  |  |
| *Varroa* mite infestation rate | Site | 5 | 25.8 | 5.16 | 6.06 | <0.0001 |
|  | Year | 2 | 37.3 | 18.67 | 21.95 | <0.0001 |
|  | Site*Year | 10 | 59.6 | 5.96 | 7.01 | <0.0001 |
|  | Residuals | 393 | 334.4 | 0.851 |  |  |
| *Nosema* spp. | Site | 5 | 4.0x10^12^ | 8.0x10^11^ | 7.13 | <0.0001 |
|  | Year | 2 | 2.6x10^11^ | 1.3x10^11^ | 1.19 | 0.31 |
|  | Site*Year | 10 | 5.0x10^12^ | 5.0x10^11^ | 4.50 | <0.0001 |
|  | Residuals | 393 | 4.4x10^13^ | 1.1x10^11^ |  |  |
| Acute bee paralysis virus | Site | 5 | 607.8 | 121.57 | 12.02 | <0.0001 |
|  | Year | 2 | 5.7 | 2.86 | 0.28 | 0.76 |
|  | Site*Year | 10 | 507.1 | 50.71 | 5.01 | <0.0001 |
|  | Residuals | 90 | 910.6 | 10.12 |  |  |
| Black queen cell virus | Site | 5 | 377.7 | 75.53 | 4.07 | 0.002 |
|  | Year | 2 | 3.9 | 1.95 | 0.11 | 0.90 |
|  | Site*Year | 10 | 492.6 | 49.26 | 2.65 | 0.007 |
|  | Residuals | 90 | 1671.2 | 18.57 |  |  |
| Chronic bee paralysis virus | Site | 5 | 19.6 | 3.92 | 0.31 | 0.91 |
|  | Year | 2 | 64.5 | 32.23 | 2.55 | 0.08 |
|  | Residuals | 100 | 1263.2 | 12.63 |  |  |
| Deformed wing virus | Site | 5 | 842 | 168.4 | 3.06 | 0.01 |
|  | Year | 2 | 3485 | 1742.7 | 31.68 | <0.0001 |
|  | Site*Year | 10 | 2659 | 265.9 | 4.83 | <0.0001 |
|  | Residuals | 90 | 4952 | 55.0 |  |  |
| Israeli acute paralysis virus | Site | 5 | 242.7 | 48.54 | 2.04 | 0.08 |
|  | Year | 2 | 71.8 | 35.88 | 1.51 | 0.23 |
|  | Residuals | 100 | 2379.2 | 23.79 |  |  |
| Kashmir bee virus | Site | 5 | 67.7 | 13.55 | 1.33 | 0.26 |
|  | Year | 12 | 129.4 | 64.70 | 6.35 | 0.003 |
|  | Residuals | 100 | 1018.2 | 10.18 |  |  |
| Sacbrood virus | Site | 5 | 228 | 45.64 | 0.80 | 0.55 |
|  | Year | 2 | 592 | 296 | 5.19 | 0.007 |
|  | Residuals | 100 | 5706 | 57.06 |  |  |
| Abdominal Lipids | Site | 5 | 0.02 | 0.003 | 2.83 | 0.02 |
|  | Year | 2 | 0.12 | 0.06 | 54.59 | <0.0001 |
|  | Site*Year | 10 | 0.05 | 0.005 | 4.70 | <0.0001 |
|  | Residuals | 90 | 0.10 | 0.001 |  |  |
| Hypopharyngeal Gland Size | Site | 5 | 0.001 | 0.0001 | 2.67 | 0.03 |
|  | Year | 2 | 0.002 | 0.001 | 19.59 | <0.0001 |
|  | Site*Year | 10 | 0.004 | 0.0004 | 7.38 | <0.0001 |
|  | Residuals | 90 | 0.005 | 0.0001 |  |  |
| *Insulin-like Peptide 1* | Site | 5 | 51.2 | 10.24 | 4.82 | 0.001 |
|  | Year | 2 | 521.3 | 260.63 | 122.69 | <0.0001 |
|  | Site*Year | 10 | 41.1 | 4.11 | 1.93 | 0.05 |
|  | Residuals | 89 | 189.1 | 2.12 |  |  |
| *Vitellogenin* | Site | 5 | 55.96 | 11.19 | 4.26 | 0.001 |
|  | Year | 2 | 159.56 | 79.78 | 30.40 | <0.0001 |
|  | Residuals | 100 | 262.49 | 2.62 |  |  |
| *Abaecin* | Site | 5 | 137.0 | 27.40 | 5.07 | 0.0003 |
|  | Year | 2 | 115.9 | 57.93 | 10.72 | <0.0001 |
|  | Residuals | 100 | 540.5 | 5.41 |  |  |
| *Defensin 1* | Site | 5 | 67.15 | 13.43 | 4.66 | 0.001 |
|  | Year | 2 | 148.44 | 74.22 | 25.77 | <0.0001 |
|  | Site*Year | 10 | 78.56 | 7.86 | 2.73 | 0.006 |
|  | Residuals | 90 | 259.22 | 2.88 |  |  |
| *Hymenoptaecin* | Site | 5 | 89.06 | 17.81 | 5.82 | 0.0001 |
|  | Year | 2 | 151.80 | 75.90 | 24.81 | <0.0001 |
|  | Site*Year | 10 | 164.88 | 16.49 | 5.39 | <0.0001 |
|  | Residuals | 90 | 275.30 | 3.06 |  |  |
| *Lysozyme 2* | Site | 5 | 22.5 | 4.51 | 2.81 | 0.02 |
|  | Year | 2 | 586.2 | 293.1 | 182.45 | <0.0001 |
|  | Residuals | 100 | 160.6 | 1.61 |  |  |
