# Supplementary material for: Linking Measures of Colony and Individual Honey Bee Health to Survival among Apiaries Exposed to Varying Agricultural Land Use
Source: PLoS One. 2016 Mar 30;11(3):e0152685. doi: 10.1371/journal.pone.0152685 (PMC4814072; doi:10.1371/journal.pone.0152685)
Supplement: S3 Table — Comparisons shown are those that were significant at p < 0.05 within a given date. (DOCX) [file pone.0152685.s003.docx]

**S3 Table. Tukey HSD comparisons of site x year interactions.** Comparisons shown are those that were significant at p < 0.05 within a given date.

| Measure |  | Comparisons (month.year.site) | Mean Difference | 95% CI | |
| --- | --- | --- | --- | --- | --- |
|  |  |  |  | Lower | Upper |
| Number combs of adult bees |  | Jul.2010.A vs. B | 5.07 | 2.30 | 7.83 |
|  |  | Jul.2010.D vs. B | 4.25 | 1.49 | 7.02 |
|  |  | Jul.2010.E vs. B | 5.61 | 2.84 | 8.37 |
|  |  | Jul.2010.F vs. B | 5.02 | 2.26 | 7.79 |
|  |  | Sep.2010.A vs. B | 3.17 | 0.25 | 6.08 |
|  |  | Sep.2010.D vs. E | -3.64 | -6.62 | -0.66 |
|  |  | Sep.2010.E vs. B | 4.00 | 0.98 | 7.01 |
| Number combs of pupating brood |  | Sep.2010.D vs. C | -0.33 | -0.66 | -0.01 |
|  |  | Sep.2011.A vs. F | -0.39 | -0.73 | -0.06 |
|  |  | Sep.2011.D vs. F | -0.60 | -0.94 | -0.25 |
|  |  | Sep.2011.E vs. F | -0.60 | -0.93 | -0.26 |
|  |  | Sep.2011.F vs. B | 0.63 | 0.30 | 0.97 |
|  |  | Sep.2011.F vs. C | 0.75 | 0.41 | 1.09 |
|  |  | Sep.2012.A vs. C | 0.53 | 0.21 | 0.85 |
|  |  | Sep.2012.A vs. E | 0.38 | 0.06 | 0.71 |
|  |  | Jan.2011.A vs. C | 0.12 | 0.04 | 0.20 |
|  |  | Jan.2011.D vs. C | 0.10 | 0.02 | 0.19 |
|  |  | Jan.2011.A vs. B | 0.13 | 0.05 | 0.21 |
|  |  | Jan.2011.D vs. B | 0.11 | 0.03 | 0.19 |
| *Varroa* mite infestation percent |  | May.2011.F vs. A | 0.23 | 0.05 | 0.41 |
|  |  | May.2011.F vs. B | 0.23 | 0.05 | 0.41 |
|  |  | May.2011.F vs. C | 0.23 | 0.05 | 0.41 |
|  |  | May.2011.F vs. D | 0.23 | 0.05 | 0.41 |
|  |  | May.2011.F vs. E | 0.23 | 0.05 | 0.41 |
|  |  | Aug.2012.A vs. E | -1.59 | -2.98 | -0.20 |
|  |  | Aug.2012.C vs. B | -1.68 | -3.07 | -0.29 |
|  |  | Aug.2012.C vs. D | -1.99 | -3.38 | -0.61 |
|  |  | Aug.2012.C vs. E | -2.41 | -3.80 | -1.03 |
|  |  | Aug.2012.C. vs. F | -1.88 | -3.30 | -0.46 |
|  |  | Sep.2012.A vs. B | -1.03 | -1.97 | -0.08 |
|  |  | Sep.2012.A vs. E | -1.55 | -2.49 | -0.62 |
|  |  | Sep.2012.B vs. C | 1.49 | 0.55 | 2.44 |
|  |  | Sep.2012.D vs. B | -1.41 | -2.37 | -0.46 |
|  |  | Sep.2012.D vs. E | -1.94 | -2.88 | -0.99 |
|  |  | Sep.2012.E vs. C | 2.02 | 1.08 | 2.95 |
|  |  | Sep.2012.E vs. F | 1.75 | 0.78 | 2.72 |
|  |  | Sep.2012.F vs. B | -1.22 | -2.20 | -0.25 |
|  |  | Nov.2012.A vs. C | 1.40 | 0.21 | 2.59 |
|  |  | Nov.2012.A vs. B | 1.40 | 0.19 | 2.62 |
|  |  | Nov.2012.D vs. E | -1.77 | -3.00 | -0.54 |
|  |  | Nov.2012.E vs. B | 2.36 | 1.12 | 3.61 |
|  |  | Nov.2012.E vs. C | 2.36 | 1.15 | 3.58 |
| Varroa mite infestation percent |  | Nov.2012.F vs. B | 1.75 | 0.49 | 3.01 |
|  |  | Nov.2012.F vs. C | 1.75 | 0.52 | 2.98 |
|  |  | Jan.2012.A vs. C | 1.28 | 0.003 | 2.55 |
|  |  | Jan.2012.D vs. E | -1.51 | -2.86 | -0.16 |
|  |  | Jan.2012.D vs. F | -1.49 | -2.82 | -0.16 |
|  |  | Jan.2012.E vs. B | 2.04 | 0.64 | 3.43 |
|  |  | Jan.2012.E vs. C | 2.10 | 0.76 | 3.43 |
|  |  | Jan.2012.F vs. B | 2.02 | 0.64 | 3.39 |
|  |  | Jan.2012.F vs. C | 2.08 | 0.76 | 3.39 |
| *Nosema* spores per 100 bees (x10^3^) |  | May.2010.F vs. A | -2277 | -3988 | -566 |
|  |  | May.2010.F vs. B | -2408 | -4119 | -697 |
|  |  | May.2010.F vs. C | -1773 | -3484 | -62 |
|  |  | May.2010.F vs. D | -2548 | -4259 | -837 |
|  |  | May.2010.F vs. E | -2406 | -4117 | -695 |
|  |  | Aug.2011.B vs. E | -2138 | -4173 | -102 |
|  |  | Aug.2011.C vs. A | 4118 | 2060 | 6176 |
|  |  | Aug.2011.C vs. B | 4430 | 2373 | 6488 |
|  |  | Aug.2011.C vs. D | 3203 | 1146 | 5261 |
|  |  | Aug.2011.C vs. E | 2293 | 235 | 4351 |
|  |  | Aug.2011.C vs. F | 4244 | 2141 | 6347 |
|  |  | Sep.2011.C vs. A | 689 | 343 | 1035 |
|  |  | Sep.2011.C vs. B | 627 | 280 | 973 |
|  |  | Sep.2011.C vs. D | 625 | 271 | 979 |
|  |  | Sep.2011.C vs. E | 635 | 289 | 981 |
|  |  | Sep.2011.C vs. F | 710 | 352 | 1068 |
|  |  | Nov.2011.C vs. A | 3040 | 2216 | 3865 |
|  |  | Nov.2011.C vs. B | 2736 | 1912 | 3561 |
|  |  | Nov.2011.C vs. D | 3050 | 2207 | 3893 |
|  |  | Nov.2011.C vs. E | 3034 | 2201 | 3867 |
|  |  | Nov.2011.C vs. F | 3115 | 2262 | 3967 |
| Hypopharyngeal gland size (mm) |  | Sep.2010.B vs. A | 0.02 | 0.004 | 0.03 |
|  |  | Sep.2010.B vs. D | 0.02 | 0.01 | 0.04 |
|  |  | Sep.2010.B vs. E | 0.03 | 0.01 | 0.04 |
|  |  | Sep.2010.C vs. E | 0.02 | 0.003 | 0.03 |
|  |  | Sep.2010.E vs. F | -0.02 | -0.04 | -0.005 |
| Proportion abdominal lipids |  | Aug.2010.B vs. F | 0.08 | 0.02 | 0.14 |
|  |  | Aug.2010.C vs. F | 0.06 | 0.0002 | 0.12 |
|  |  | Aug.2010.E vs. F | 0.08 | 0.02 | 0.14 |
|  |  | Aug.2011.A vs. D | 0.06 | 0.003 | 0.12 |
|  |  | Aug.2011.C vs. D | 0.06 | 0.001 | 0.12 |
|  |  | Aug.2011.F vs. D | 0.1 | 0.04 | 0.16 |
|  |  | Aug.2011.F vs. E | 0.07 | 0.01 | 0.13 |
|  |  | Sep.2010.A vs. B | 0.09 | 0.02 | 0.16 |
|  |  | Sep.2010.A vs. F | 0.08 | 0.01 | 0.15 |
| Proportion abdominal lipids |  | Sep.2010.B vs. C | -0.08 | -0.15 | -0.02 |
|  |  | Sep.2010.B vs. D | -0.08 | -0.15 | -0.02 |
|  |  | Sep.2010.B vs. E | -0.07 | -0.14 | -0.001 |
|  |  | Sep.2010.C vs. F | 0.07 | 0.004 | 0.14 |
|  |  | Sep.2010.D vs. F | 0.08 | 0.005 | 0.14 |
|  |  | Mar.2011.C vs. F | -0.08 | -0.15 | -0.01 |
