# Supplementary material for: Linking Measures of Colony and Individual Honey Bee Health to Survival among Apiaries Exposed to Varying Agricultural Land Use
Source: PLoS One. 2016 Mar 30;11(3):e0152685. doi: 10.1371/journal.pone.0152685 (PMC4814072; doi:10.1371/journal.pone.0152685)
Supplement: S4 Table — Colony level expression: viruses and reference gene, RPS5; Individual bee level expression: nutritional and immune genes and reference gene, ß-Actin. (DOCX) [file pone.0152685.s004.docx]

**S4 Table. Primer sequences used for gene expression analysis.** Colony level expression: viruses and reference gene, RPS5; Individual bee level expression: nutritional and immune genes and reference gene, ß-Actin.

|  | Type | Direction | Primer Sequence |
| --- | --- | --- | --- |
| RPS5 | Reference | forward | AATTATTTGGTCGCTGGAATTG |
|  |  | reverse | TAACGTCCAGCAGAATGTGGTA |
| ABPV | Viral | forward | ACCGACAAAGGGTATGATGC |
|  |  | reverse | CTTGAGTTTGCGGTGTTCCT |
| BQCV | Viral | forward | TTTAGAGCGAATTCGGAAACA |
|  |  | reverse | GGCGTACCGATAAAGATGGA |
| CBPV | Viral | forward | CAAAATCAACGAGCCAATCA |
|  |  | reverse | AGTGTGAGGATCACCGGAAC |
| DWV | Viral | forward | GAGATTGAAGCGCATGAACA |
|  |  | reverse | TGAATTCAGTGTCGCCCATA |
| IAPV | Viral | forward | CCATGCCTGGCGATTCAC |
|  |  | reverse | CTGAATAATACTGTGCGTATC |
| KBV | Viral | forward | TGAACGTCGACCTATTGAAAAA |
|  |  | reverse | TCGATTTTCCATCAAATGAGC |
| SBV | Viral | forward | GGGTCGAGTGGTACTGGAAA |
|  |  | reverse | ACACAACACTCGTGGGTGAC |
| *β-Actin* | Reference | forward | TTGTATGCCAACACTGTCCTTT |
|  |  | reverse | TGGCGCGATGATCTTAATTT |
| *Insulin-like Peptide 1* | Nutrition | forward | GCTCAGGCTGTGCTCGAAAAGT |
|  |  | reverse | CGTTGTATCCACGACCCTTGC |
| *Vitellogenin* | Nutrition | forward | AGTTCCGACCGACGACG |
|  |  | reverse | TTCCCTCCCACGGAGTCC |
| *Abaecin* | Immunity | forward | CAGCATTCGCATACGTACCA |
|  |  | reverse | GACCAGGAAACGTTGGAAAC |
| *Defensin 1* | Immunity | forward | TGCGCTGCTAACTGTCTCAG |
|  |  | reverse | AATGGCACTTAACCGAAACG |
| *Hymenoptaecin* | Immunity | forward | CTCTTCTGTGCCGTTGCATA |
|  |  | reverse | GCGTCTCCTGTCATTCCATT |
| *Lysozyme 2* | Immunity | forward | CCAAATTAACAGCGCCAAGT |
|  |  | reverse | GCAATTCTTCACCCAACCAT |
