# Supplementary figures and images for: Linking Measures of Colony and Individual Honey Bee Health to Survival among Apiaries Exposed to Varying Agricultural Land Use
Source: PLoS One. 2016 Mar 30;11(3):e0152685. doi: 10.1371/journal.pone.0152685 (PMC4814072; doi:10.1371/journal.pone.0152685)

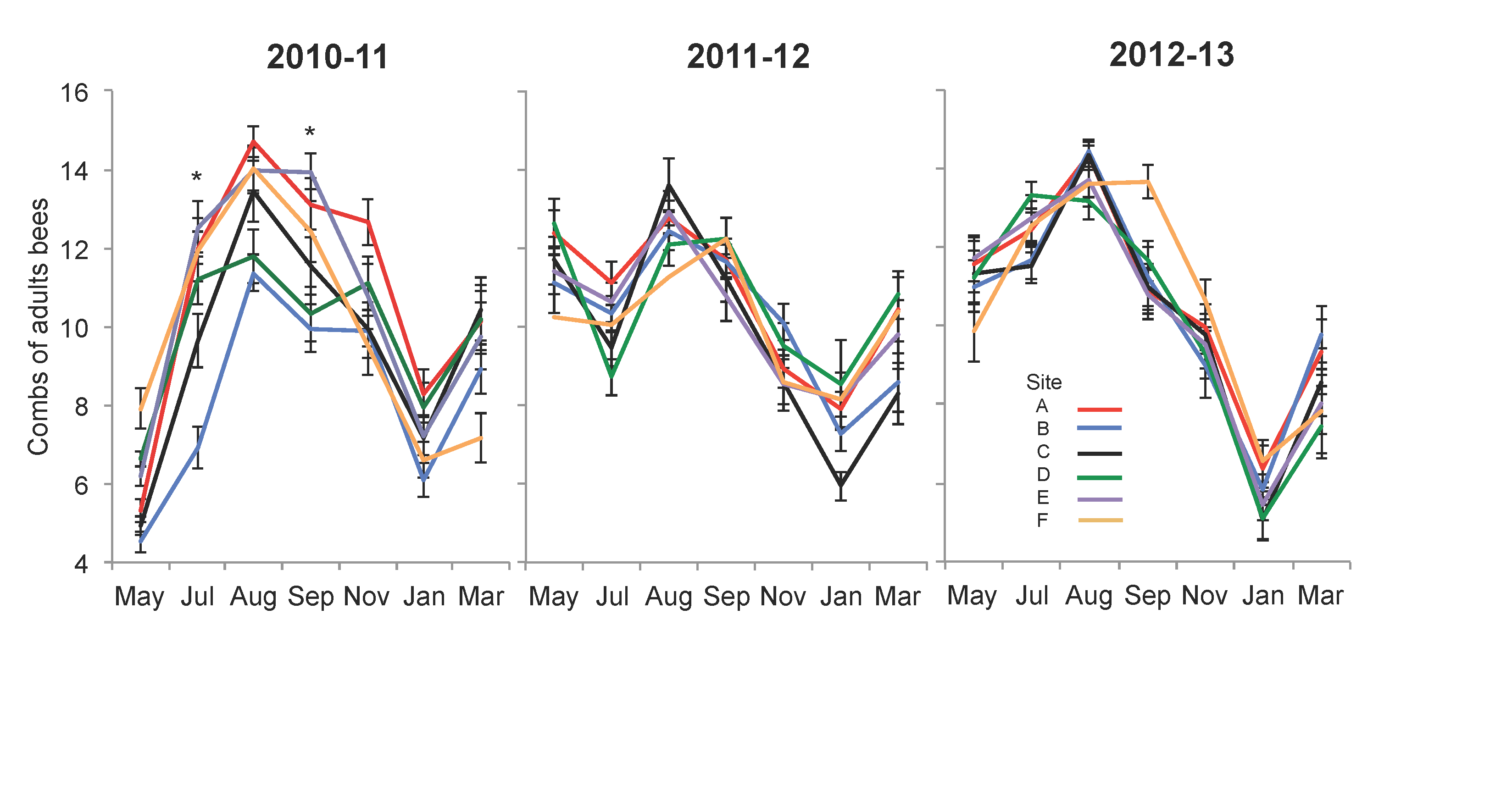

Supplement: S1 Fig — Asterisks denote significant differences among sites on a given sample date. (TIFF) [file pone.0152685.s005.tiff]

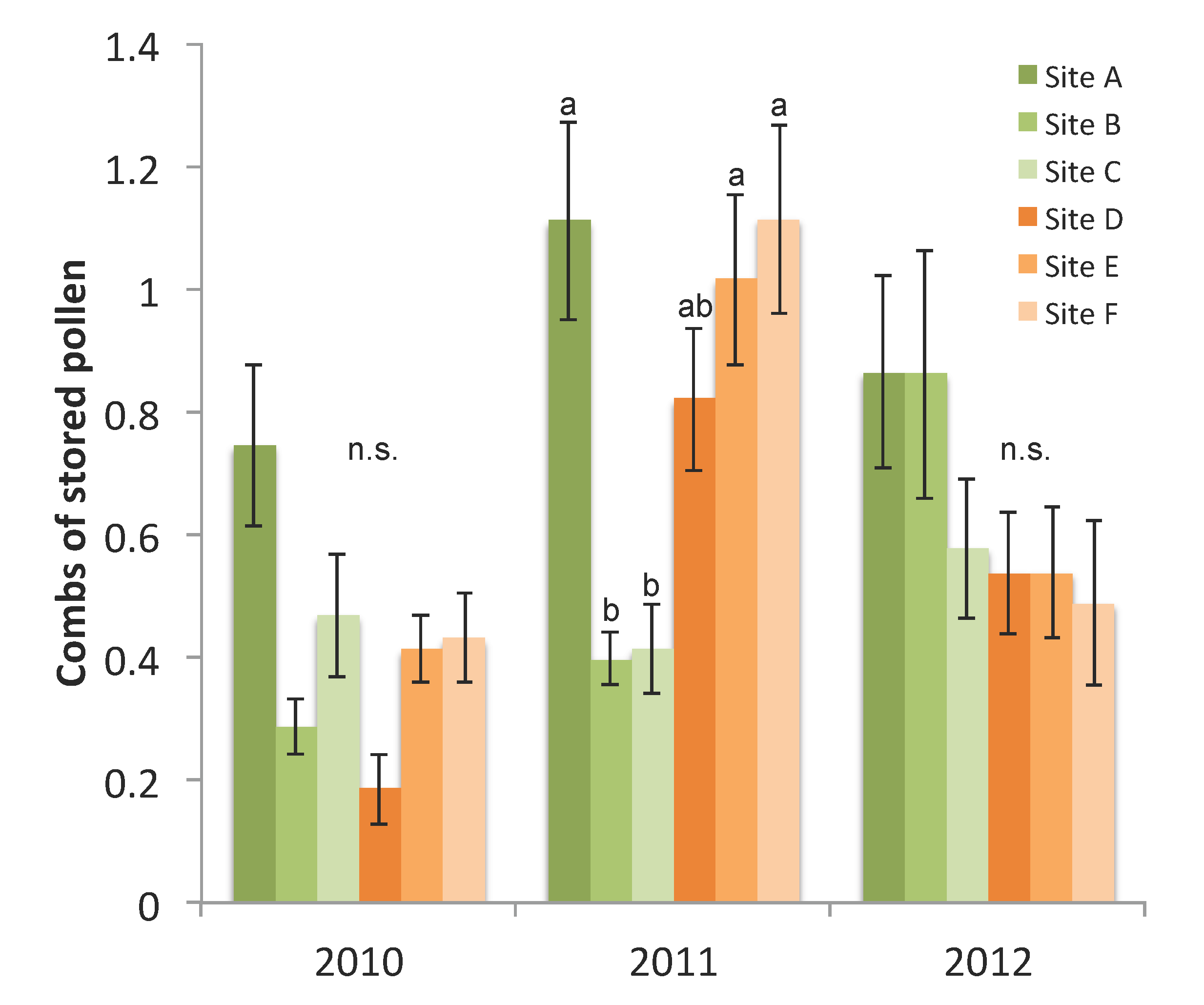

Supplement: S2 Fig — Letters denote significant differences among sites within each year. (TIFF) [file pone.0152685.s006.tiff]
